# Functionalized 3-(5-ar­yloxy-3-methyl-1-phenyl-1*H*-pyrazol-4-yl)-1-(4-substituted-phen­yl)prop-2-en-1-ones: synthetic pathway, and the structures of six examples

**DOI:** 10.1107/S2056989020005113

**Published:** 2020-04-21

**Authors:** Haruvegowda Kiran Kumar, Hemmige S. Yathirajan, Asma, Nagaraja Manju, Balakrishna Kalluraya, Ravindranath S. Rathore, Christopher Glidewell

**Affiliations:** a Department of Studies in Chemistry, University of Mysore, Manasagangotri, Mysuru-570 006, India; b Department of Studies in Chemistry, Mangalore University, Mangalagangotri, Mangalore-574199, India; c Department of Bioinformatics, School of Earth, Biological and Environmental Sciences, Central University of South Bihar, Gaya-824236, India; d School of Chemistry, University of St Andrews, St Andrews, Fife KY16 9ST, UK

**Keywords:** synthesis, substituted pyrazoles, chalcones, crystal structures, disorder, mol­ecular conformation, hydrogen boding, supra­molecular assembly

## Abstract

Five examples each of 3-(5-ar­yloxy-3-methyl-1-phenyl-1*H*-pyrazol-4-yl)-1-[4-(prop-2-yn-1-yl­oxy)phen­yl]prop-2-en-1-ones and the corresponding 1-(4-azido­phen­yl)-3-(5-ar­yloxy-3-methyl-1-phenyl-1*H*-pyrazol-4-yl)prop-2-en-1-ones have been synthesized in a highly efficient manner, starting from a common source precursor, and structures have been determined for three examples of each type. In each of 3-[5-(2-chloro­phen­oxy)-3-methyl-1-phenyl-1*H*-pyrazol-4-yl]-1-[4-(prop-2-yn-1-yl­oxy)phen­yl]prop-2-en-1-one, C_28_H_21_ClN_2_O_3_, (Ib), the isomeric 3-[5-(2-chloro­phen­oxy)-3-methyl-1-phenyl-1*H*-pyrazol-4-yl]-1-[4-(prop-2-yn-1-yl­oxy)phen­yl]prop-2-en-1-one, (Ic), and 3-[3-methyl-5-(naphthalen-2-yl­oxy)-1-phenyl-1*H*-pyrazol-4-yl]-1-[4-(prop-2-yn­yloxy)phen­yl]prop-2-en-1-one, C_32_H_24_N_2_O_3_, (Ie), the mol­ecules are linked into chains of rings, formed by two independent C—H⋯O hydrogen bonds in (Ib) and by a combination of C—H⋯O and C—H⋯π(arene) hydrogen bonds in each of (Ic) and (Ie). There are no direction-specific inter­molecular inter­actions in the structure of 1-(4-azido­phen­yl)-3-[3-methyl-5-(2-methyl­phen­oxy)-1-phenyl-1*H*-pyrazol-4-yl]prop-2-en-1-one, C_26_H_21_N_5_O_2_, (IIa). In 1-(4-azido­phen­yl)-3-[5-(2,4-di­chloro­phen­oxy)-3-methyl-1-phenyl-1*H*-pyrazol-4-yl]prop-2-en-1-one, C_25_H_17_Cl_2_N_5_O_2_, (IId), the di­chloro­phenyl group is disordered over two sets of atomic sites having occupancies 0.55 (4) and 0.45 (4), and the mol­ecules are linked by a single C—H⋯O hydrogen bond to form cyclic, centrosymmetric *R* ^2^_2_(20) dimers. Similar dimers are formed in 1-(4-azido­phen­yl)-3-[3-methyl-5-(naphthalen-2-yl­oxy)-1-phenyl-1*H*-pyrazol-4-yl]prop-2-en-1-one, C_29_H_21_N_5_O_2_, (IIe), but here the dimers are linked into a chain of rings by two independent C—H..π(arene) hydrogen bonds. Comparisons are made between the mol­ecular conformations within both series of compounds.

## Abstract 2

Two series of functionalized chalcones have been synthesized from a common family of precursors, and the structures of three examples from each series have been determined. The supra­molecular assembly, based upon C—H⋯O and C—H⋯π(arene) hydrogen bonds, is different in all of the examples examined.

## Chemical context   

**Figure 1:**
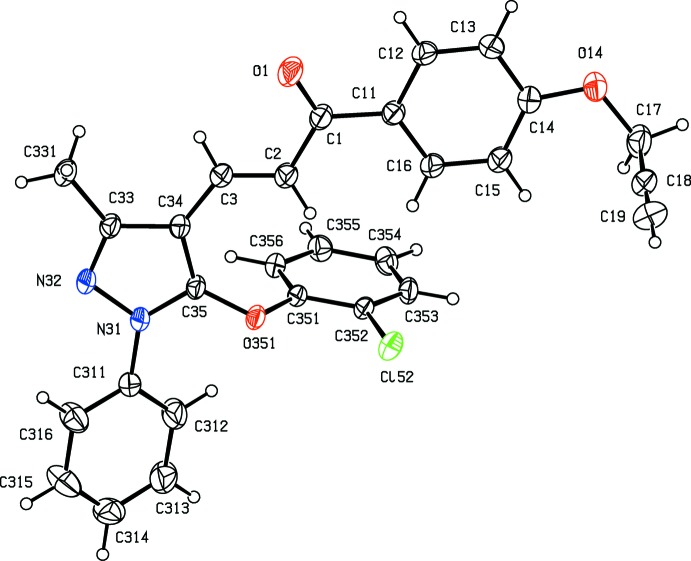
The mol­ecular structure of compound (Ib) showing the atom-labelling scheme. Displacement ellipsoids are drawn at the 30% probability level.

Chalcones, 1.3-disubstituted-prop-2-en-1-ones of type *R* ^1^COCH=CH*R* ^2^, exhibit a wide range of biological activities, particularly when they incorporate functionalized substituents; these include anti­cancer (Murthy *et al.*, 2013 ▸), anti­malarial (Mishra *et al.*, 2008 ▸; Yadav *et al.*, 2012 ▸), anti­tripanosomal (Carvalho *et al.*, 2012 ▸) and anti­viral (Sharma *et al.*, 2011 ▸) activities. With these properties in mind, we have developed a versatile and efficient route to functionalized chalcones, which are themselves the basis for further elaboration to provide a wide range of multiply substituted chalcones. Here we report the synthesis and characterization of five 3-(5-ar­yloxy-3-methyl-1-phenyl-1*H*-pyrazol-4-yl)-1-(4-(prop-2-yn-1- yloxy)phen­yl)prop-2-en-1-ones (I) and a corresponding series of five 1-(4-azido­phen­yl)-3-(3-methyl-1-phenyl-5-(ar­yloxy)-1*H*-pyrazol-4-yl)-prop-2-en-1-ones (II), together with the structures of a representative selection of three examples of each type, namely (Ib), (Ic) and (Ie) and (IIa), (IId) and (IIe) (Figs. 1 ▸–6 ▸ ▸ ▸ ▸ ▸). The compounds of types (I) and (II) were prepared using a common synthetic scheme starting from the commercially available 3-methyl-1-phenyl-1*H*-pyrazole, which was readily converted, under Vilsmaeier–Haack conditions, to the key precursor 5-chloro-3-methyl-1-phenyl-1*H*-pyrazole-4-carbaldehyde (*A*) (Fig. 7 ▸), which was then converted to a series of 5-ar­yloxy derivatives (*B*), by reaction with substituted phenols under basic conditions as previously described (Kiran Kumar *et al.*, 2019 ▸). The 5-ar­yloxy compounds (*B*) were then condensed with 1-[4-(prop-2-yn-1-yl­oxy)phen­yl]ethan-1-one to give the products (Ia)–(Ie) (Fig. 7 ▸) or with 1-(4-azido­phen­yl)ethan-1-one to give the corresponding series of products (IIa)–(IIe). Thus the synthesis of these two matched series of products (I) and (II) from common precursors, is highly efficient. The presence of the alkyne unit in the type (I) products and of the azido unit in the type (II) products means that a small library is now available for use in Huisgen-type cyclo­addition reactions to form bis­(chalcone)-substituted 1,2,3-triazoles. Such highly functionalized triazoles are an attractive synthetic target as 1,2,3-triazoles, which exhibit a very wide range of biological activity of potential medicinal values (Kharb *et al.*, 2011 ▸; Dheer *et al.*, 2017 ▸).
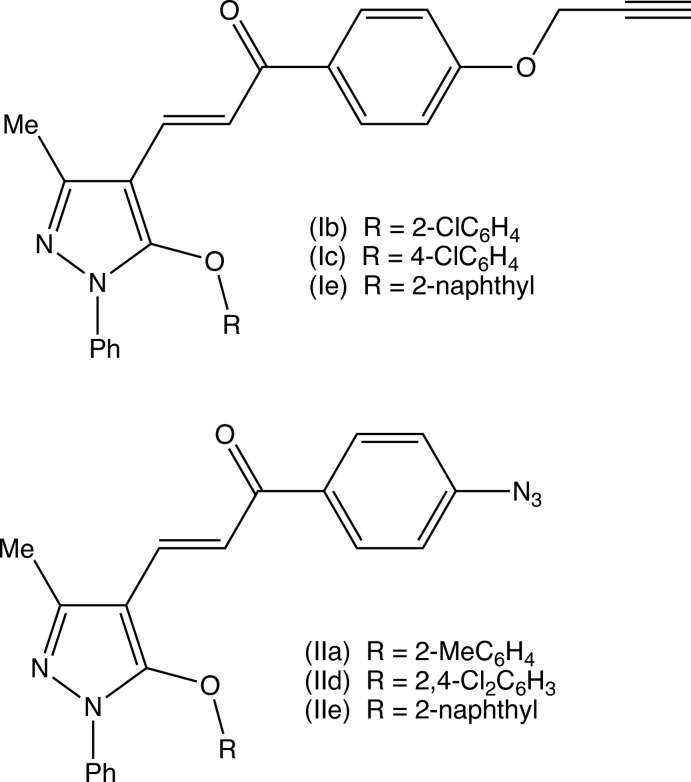


## Structural commentary   

**Figure 3:**
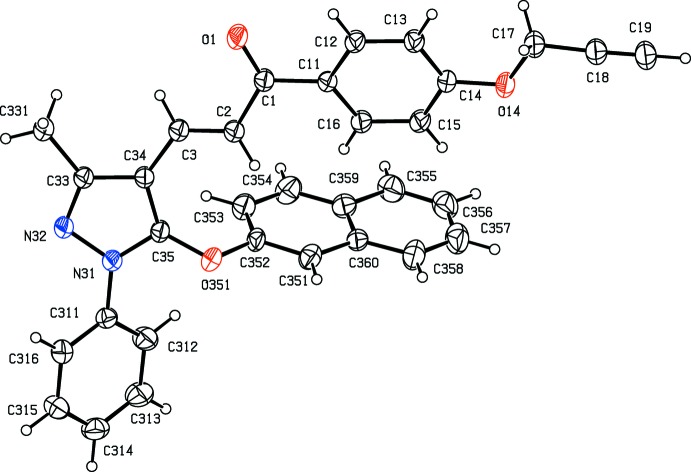
The mol­ecular structure of compound (Ie) showing the atom-labelling scheme. Displacement ellipsoids are drawn at the 30% probability level.

Compounds (Ib) and (Ic) are geometrical isomers (Figs. 1 ▸ and 2 ▸), although they are not isomorphous (Table 3 ▸). Although the constitutions of compounds (Ie) and (IIe) differ only in the identity of the small substituent at atom C14 (Figs. 3 ▸ and 6 ▸), these compounds crystallize in different space groups (Table 3 ▸).

In each of the compounds reported here, the central core of the mol­ecules, comprising the pyrazole ring and the adjacent prop-2-en-1-one unit is very nearly planar. However, the three substituents at atoms C1, N31 and C35 (Figs. 1 ▸–6 ▸ ▸ ▸ ▸ ▸) are all twisted out of the plane of the mol­ecular core, as indicated by the relevant torsional angles (Table 1 ▸). None of the mol­ecules, therefore, exhibits any inter­nal symmetry, so that all are conformationally chiral: however, the space groups confirm that they have all crystallized as conformational racemates. In each case, the reference mol­ecule was selected to be one having a positive sign for the torsional angle N32—N31—C311—C312 (Table 1 ▸).

**Figure 5:**
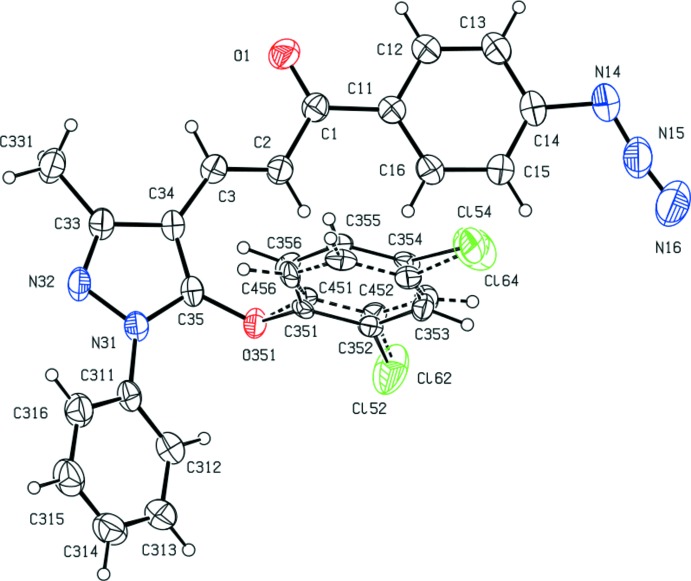
The mol­ecular structure of compound (IId) showing the atom-labelling scheme, and the disorder in the 2,4-di­chloro­phenyl group. The major disorder component is drawn using full lines and the minor disorder component is drawn using broken lines. Displacement ellipsoids are drawn at the 30% probability level.

In compound (IId), the 2,4-di­chloro­phen­oxy substituent was found to be disordered over two sets of atomic sites, having occupancies 0.55 (4) and 0.45 (4) (Fig. 5 ▸). The disorder involves slight differences in the torsional angles around the bond C35-O351 (Fig. 5 ▸), thus C34—C35—O351—C*x*51 = 78.3 (16)° when *x* = 3, and 65.0 (12)° when *x* = 4: on the other hand, the torsional angles around the bonds O351—C*x*51 (*x* = 3 or 4) are the same within experimental uncertainty, thus C35—O351—C*x*51—C*x*52 = −164 (2)° for *x* = 3 and −163.6 (18)° when *x* = 4.

The orientation of the OCH_2_CCH substituent relative to the adjacent aryl ring is different in compound (Ib), as compared with (Ic) and (Ie) (Table 1 ▸, Figs. 1 ▸–3 ▸ ▸) and similarly the orientation of the azido substituent is different in (IId), as compared with (IIa) and (IIe). In the case of the type (I) compounds, it is tempting to associate the observed differences with the different patterns of hydrogen bonding (Table 2 ▸, and Section 3, below), where atom O14 acts as an acceptor only in (Ib) while atom C19 acts as a donor in (Ic) and (Ie) but not in (Ib). However, in none of the type (II) compounds do any of the N atoms of the azido unit act as a hydrogen-bond acceptor. Hence, in these compounds, at least, the role of this substituent may be mainly that of a space filler, with the conformation adopted being that which most effectively fills any available space between the mol­ecules.

## Supra­molecular features   

The supra­molecular assembly in the structures reported here is dominated by C—H⋯O hydrogen bonds (Table 2 ▸), along with C—H⋯π(arene) hydrogen bonds in compounds (Ic), (Ie) and (IIe). However, in none of the compounds containing chloro­phen­oxy substituents, [(Ib), (Ic) and (IId)] are there any short C—Cl⋯π(arene) contacts (cf. Imai *et al.*, 2008 ▸).

**Figure 7:**
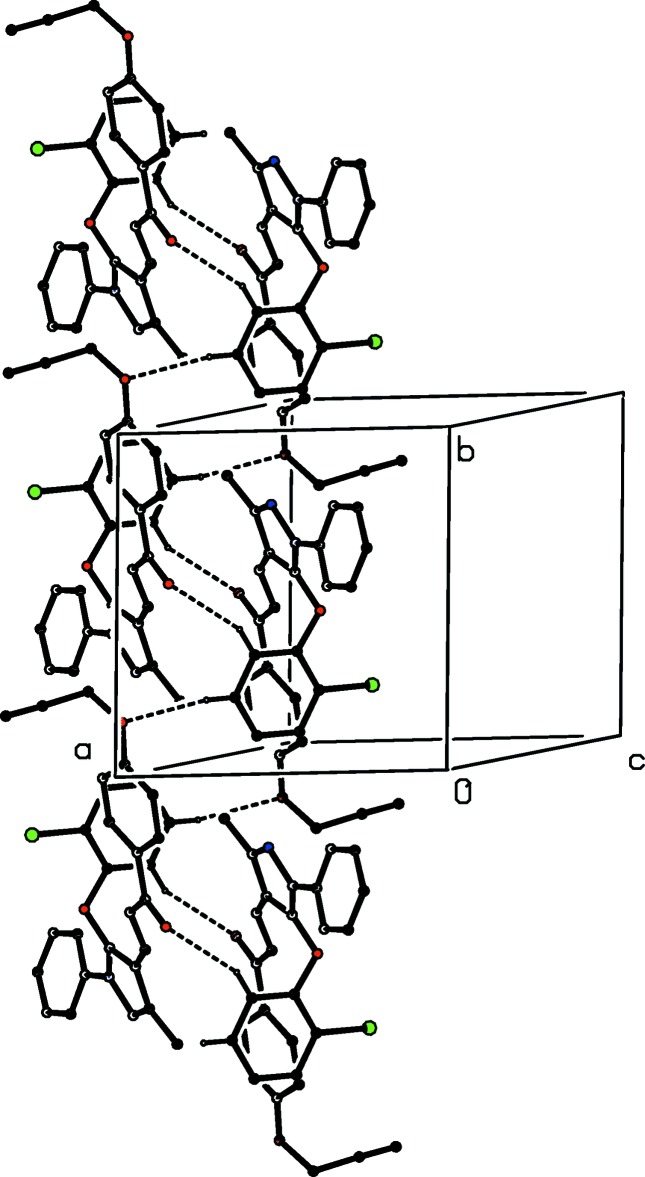
Part of the crystal structure of compound (Ib) showing the formation of a chain of centrosymmetric rings parallel to [010]. Hydrogen bonds are drawn as dashed lines and, for the sake of clarity, the H atoms not involved in the motifs shown have been omitted.

**Figure 8:**
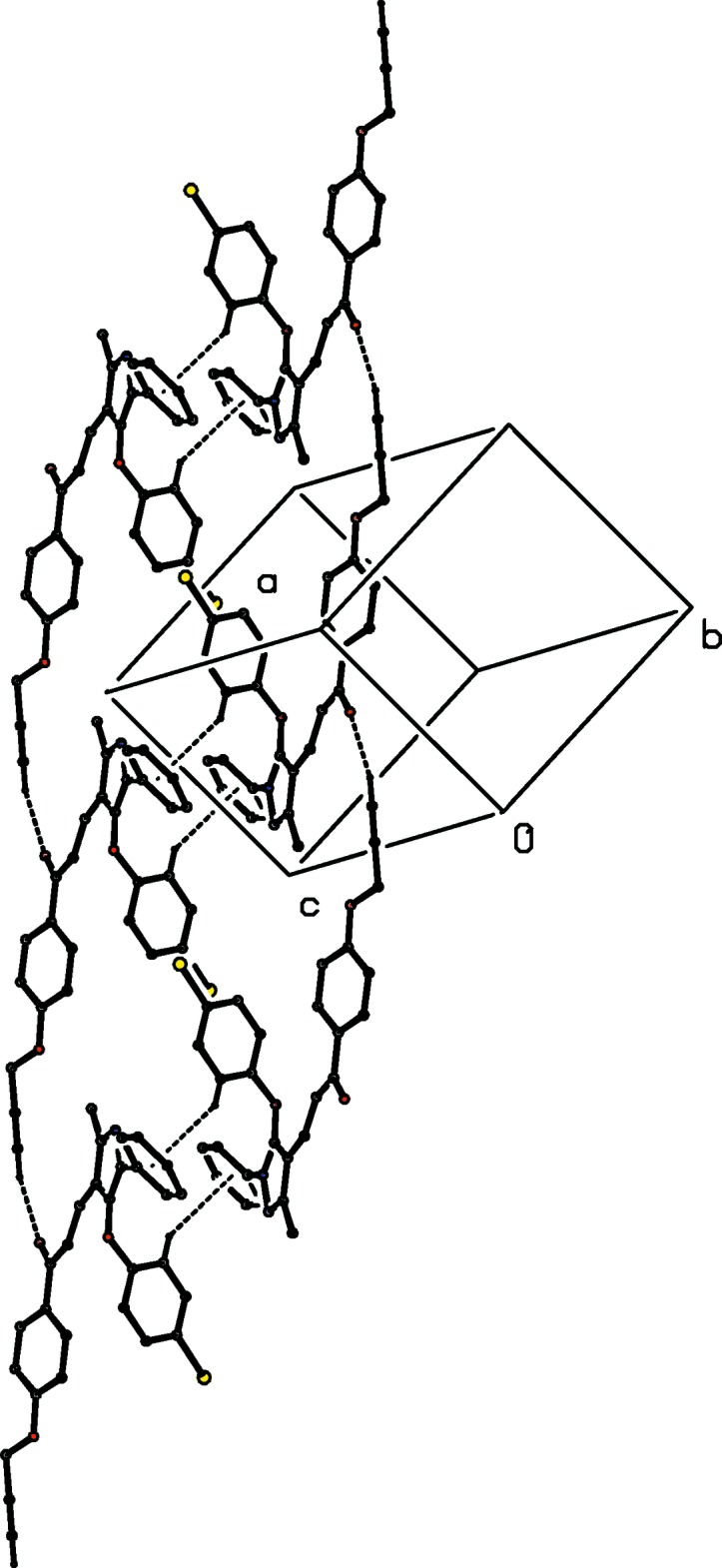
Part of the crystal structure of compound (Ic) showing the formation of a chain of rings parallel to [110]. Hydrogen bonds are drawn as dashed lines and, for the sake of clarity, the H atoms not involved in the motifs shown have been omitted.

**Figure 9:**
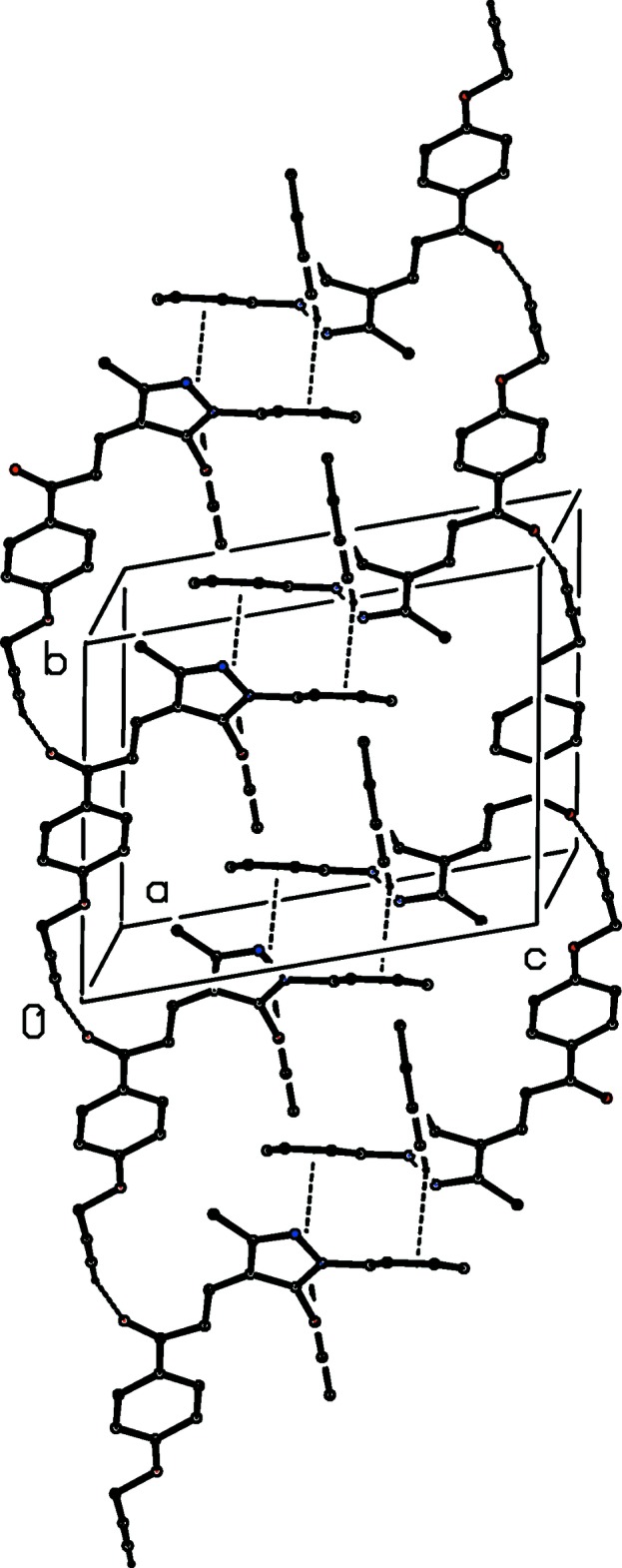
Part of the crystal structure of compound (Ie) showing the formation of a chain of rings parallel to [1
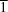
0]. Hydrogen bonds are drawn as dashed lines and, for the sake of clarity, the H atoms not involved in the motifs shown have been omitted.

There are two C—H⋯O hydrogen bonds in the structure of compound (Ib) (Table 2 ▸), and together these link the mol­ecules into a chain of centrosymmetric rings running parallel to the [010] direction, with rings of 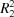
(20) type (Etter, 1990 ▸; Etter *et al.*, 1990 ▸; Bernstein *et al.*, 1995 ▸) centred at (1, *n* + 0.5, 0.5) alternating with rings of 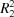
(30) type centred at (1, *n*, 0.5), where *n* represents an integer in each case (Fig. 7 ▸). In addition to an intra­molecular C—H⋯π(arene) hydrogen bond (Table 2 ▸), the structure of compound (Ic), isomeric with (Ib), contains two hydrogen bonds, one each of C—H⋯O and C—H⋯π(arene) types. The C—H⋯O hydrogen bond links mol­ecules related by translation into a *C*(11) running parallel to the [110] direction, and inversion-related pairs of such chains are linked by the C—H⋯π(arene) hydrogen bond to form a chain of rings running parallel to [110] (Fig. 8 ▸). Although there are no intra­molecular hydrogen bonds in the structure of compound (Ie), the inter­molecular hydrogen bonds (Table 2 ▸) are similar to those in compound (Ic), although the C—H⋯π(arene) inter­action involves a different donor atom; again a chain of rings is formed, but this time it runs parallel to the [1
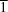
0] direction (Fig. 9 ▸).

**Figure 10:**
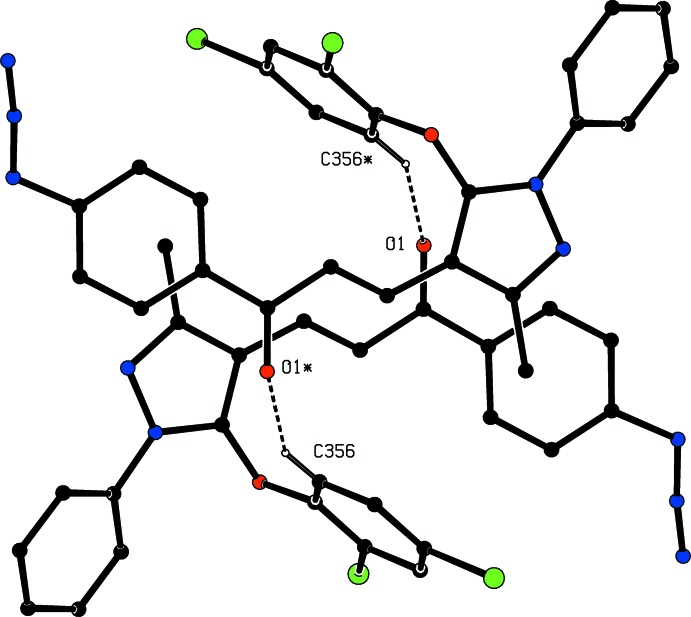
Part of the crystal structure of compound (IId) showing the formation of a centrosymmetric dimer. Hydrogen bonds are drawn as dashed lines and, for the sake of clarity, the unit-cell outline, the minor disorder component and the H atoms not involved in the motifs shown have been omitted. The atoms marked with an asterisk (*) are at the symmetry position (1 − *x*, 2 − *y*, 1 − *z*).

**Figure 11:**
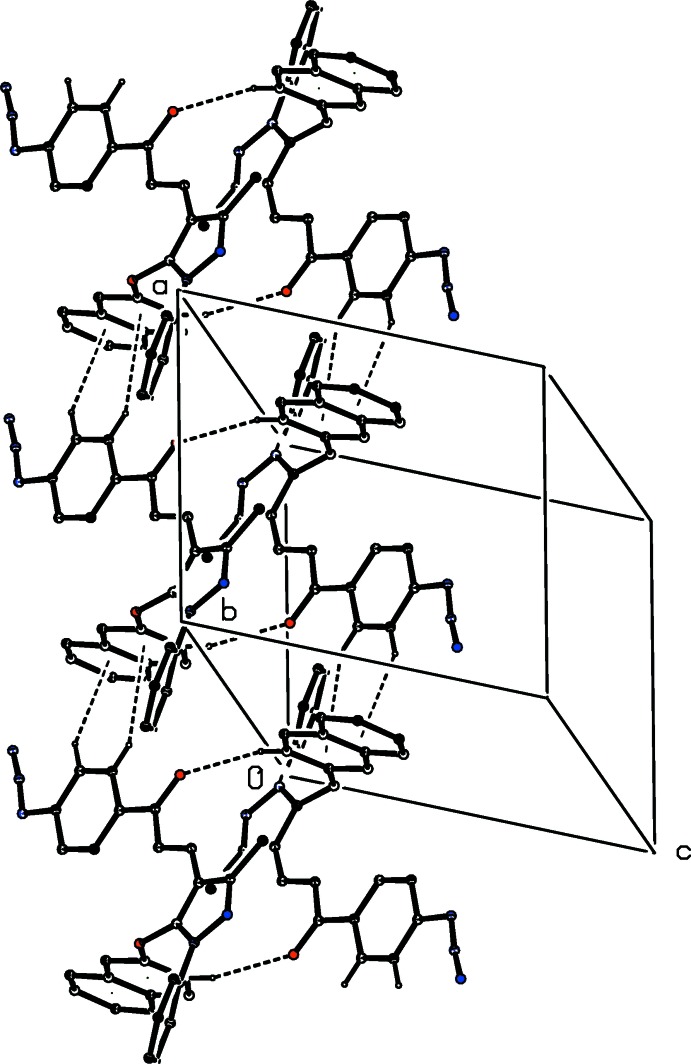
Part of the crystal structure of compound (IIe) showing the formation of a chain of rings parallel to [100]. Hydrogen bonds are drawn as dashed lines and, for the sake of clarity, the H atoms not involved in the motifs shown have been omitted.

The only direction-specific inter­molecular contact in compound (IIa) involves a methyl group. Because such groups CH_3_—*E* generally undergo rapid rotation about the C—*E* bonds, even in the solid state (Riddell & Rogerson, 1996 ▸, 1997 ▸), particularly when, as here, the methyl group is bonded to a unit having local *C*_2*v*_ (*mm*2) symmetry, when the rotational barrier is particularly low (Tannenbaum *et al.*, 1956 ▸; Naylor & Wilson, 1957 ▸). Accordingly, such a contact is not regarded as structurally significant. There is a single C—H⋯O hydrogen bond in the structure of compound (IId), with fairly similar dimensions for each of the two disorder components. Hence it is necessary to consider only the major disorder component, where the inversion-related pairs of mol­ecules are linked into cyclic, centrosymmetric 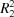
(20) dimers (Fig. 10 ▸). In the structure of compound (IIe), inversion-related pairs of mol­ecules are linked by paired C—H⋯O hydrogen bonds to form cyclic, centrosymmetric 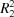
(20) dimers, which in turn are linked into a chain of rings running parallel to the [100] direction (Fig. 11 ▸) by the combined action of two C—H⋯π(arene) hydrogen bonds, which utilize both rings of the 2-naphthyl substituent as the acceptors.

Thus, the supra­molecular assembly in the isomeric pair of compounds (Ib) and (Ic) is different in terms of the hydrogen bonds involved (Table 2 ▸), although chains of rings, different in each case, are found in all three of the type (I) compounds. Amongst the type (II) compounds, (IIa) and (IId) exhibit either no direction-specific inter­molecular inter­actions, as in (IIa), or finite, zero-dimensional aggregation, as in (IId). In (IIe), a chain of rings is again found, but different from those in any of the type (I) series, although the 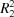
(20) motif can be identified in each of (Ib), (IId) and (IIe).

## Database survey   

**Figure 12:**
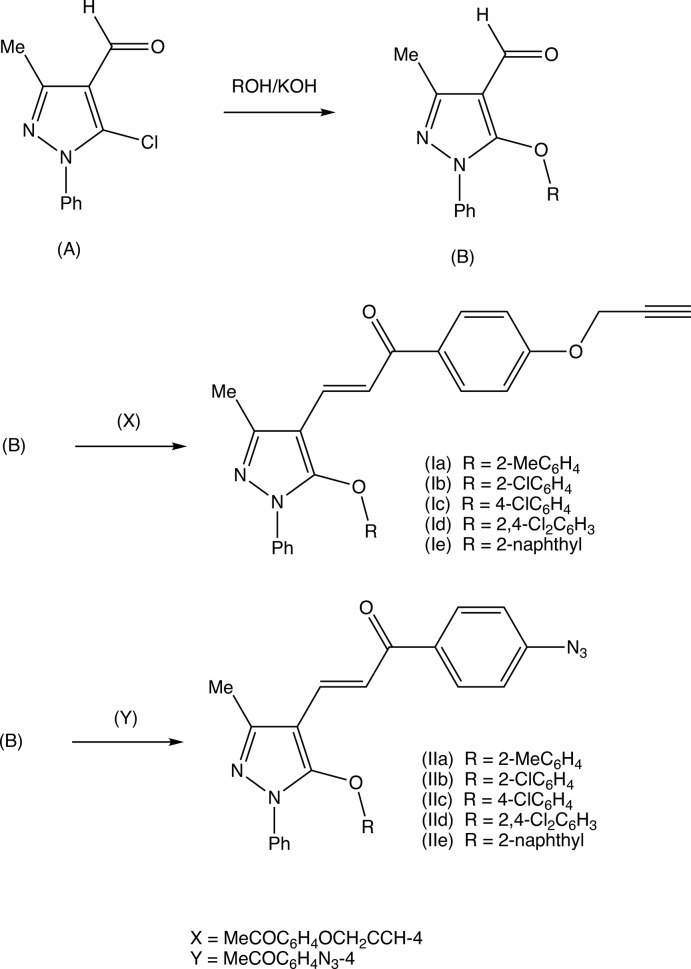
The synthetic pathway used for the preparation of compounds (Ia)–(Ie) and (IIa)–(IIe).

The structures have recently been reported (Vinutha *et al.*, 2014 ▸; Glidewell *et al.*, 2019 ▸; Kiran Kumar *et al.*, 2019 ▸) of a number of carbaldehyde precursors of type (*B*) (Fig. 12 ▸), including examples in which *R* = 2-chloro­phenyl, 4-chloro­phenyl and 2-naphthyl, *i.e.* the direct precursors for compounds (Ib), (Ic), (Ie), (IIb), (IIc) and (IIe). Structures have also been reported (Cuartas *et al.*, 2017 ▸) for both an amino analogue of (*B*), namely 5-[benz­yl(meth­yl)amino]-3-methyl-1-phenyl-1*H*-pyrazole-4-carbaldehyde and of the chalcone derived from this by condensation with 4-bromo­benzaldehyde; for the 5-(*N*-methyl­piperazino) analogue (Sunitha *et al.*, 2016 ▸) and for the 5-piperidino analogue (Kiran Kumar, 2019 ▸). Finally, we note the structures of two isostructural 3-(5-ar­yloxy-3-methyl-1-phenyl-1*H*-pyrazole-1-yl)-1-thio­phen-2-yl)prop-2-en-1-ones, both of which exhibit disorder in the orientation of the thio­phene unit (Shaibah *et al.*, 2020 ▸).

## Synthesis and crystallization   

For the preparation of the prop-2-yn-1-yl compounds (I), a solution of potassium hydroxide (0.31g, 5.7 mmol) in ethanol (30 ml) was added to a mixture of the appropriate inter­mediate of type (B, Fig.7), 5.7 mmol) and 4-(prop-2-yn-1-yl­oxy)aceto­phenone (1.0 g, 5.7 mmol) in ethanol (30 ml). The mixtures were then stirred at ambient temperature for 4 h, after which time, TLC indicated that the reactions were complete. The solid products were then collected by filtration, washed with water, dried in air and recrystallized from an ethanol–di­methyl­formamide (initial composition 3:1, *v*/*v*). Compound (Ia). Yield 82%, m.p. 551 K. IR (cm^−1^) 3228 (alkyne C-H), 2312 (alkyne C-C), 1672 (C=O), 1578 (C=N). MS (*m*/*z*) 449 (M+1)^+^. Compound (Ib). Yield 67%, m.p. 438 K. IR (cm^−1^) 3230 (alkyne C—H), 2352 (alkyne C—C), 1661 (C=O), 1581 (C=N). MS (*m*/*z*) 469 (*M*+1)^+^. Compound (Ic). Yield 77%, m.p. 406–407 K. IR (cm^−1^) 3234 (alkyne C-H), 2356 (alkyne C—C), 1668 (C=O), 1576 (C=N). MS (*m*/*z*) 469 (*M*+1)^+^. Compound (Id). Yield 65%, m.p. 485–486 K. IR (cm^−1^) 3237 (alkyne C—H), 2342 (alkyne C—C), 1676 (C=O), 1559 (C=N). MS (*m*/*z*) 469 (*M*+1)^+^. Compound (Ie). Yield 69%, m.p. 447–449 K. IR (cm^−1^) 3227 (alkyne C—H), 2360 (alkyne C—C), 1654 (C=O), 1588 (C=N). MS (*m*/*z*) 485 (*M*+1)^+^. For the preparation of the azido compounds (II), a solution of potassium hydroxide (0.34g, 6.2 mmol) in ethanol (30 ml) was added to a solution of 4-azido­aceto­phenone (1.0 g, 6.2 mmol) in ethanol (30 ml). The appropriate inter­mediate (*B*) (6.2 mmol) was then added and the mixtures were then stirred for 30 min, after which time TLC indicated that the reactions were complete. The solid products were then collected by filtration, washed with water, dried in air and recrystallized from an ethanol–di­methyl­formamide (initial composition 3:1, *v*/*v*). Compound (IIa). Yield 96%, m.p. 385–387 K. IR (cm^−1^) 2359 (azide), 1650 (C=O), 1592 (C=N). MS (*m*/*z*) 436 (*M*+1)^+^. Compound (IIb). Yield 74%, m.p. 394–396 K. IR (cm^−1^) 2355 (azide), 1674 (C=O), 1561 (C=N). MS (*m*/*z*) 456 (*M*+1)^+^. Compound (IIc). Yield 79%, m.p. 425–427 K. IR (cm^−1^) 2351 (azide), 1671 (C=O), 15612 (C=N). MS (*m*/*z*) 456 (*M*+1)^+^. Compound (IId). Yield 70%, m.p. 505 K. IR (cm^−1^) 2349 (azide), 1656 (C=O), 1592 (C=N). MS (*m*/*z*) 490 (*M*+1)^+^. Compound (IIe). Yield 74%, m.p. 489–490 K. IR (cm^−1^) 2354 (azide), 1676 (C=O), 1565 (C=N). MS (*m*/*z*) 472 (*M*+1)^+^. Crystals of compounds (Ib), (Ic), (Ie), (IIa), (IId) and (IIe) which were suitable for single-crystal X-ray diffraction were selected directly from the prepared samples: despite repeated efforts, no crystal suitable for single-crystal X-ray diffraction have yet been obtained for compounds (Ia), (Id), (IIb) or (IIc).

## Refinement   

Crystal data, data collection and refinement details are summarized in Table 3 ▸. For a number of the structures, [(Ie), (IIa), (IId) and (IIe)], the diffraction data at values of θ > 25° were uniformly of very indifferent quality, particular in terms of the symmetry-equivalent reflections. This is probably a consequence of the indifferent crystal quality, exemplifying the general difficulty within the series (I) and (II) of growing crystals suitable for single-crystal X-ray diffraction (*cf.* Section 5, above). These higher-angle reflections were therefore rejected during the data-reduction process: we note also that the intensity statistics indicated that very few of these reflections were likely to be labelled as observed for compounds (Ie), (IIa), (IId) and (IIe). A number of low-angle reflections for (Ib) and (Ie) were also discarded at this stage because of attenuation by the beam stop. Some further low-angle reflections that had been attenuated by the beam stop were omitted from the data sets before the final refinements, thus: for (Ib) (101), (110), (002), (202) and (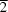
02); for (Ic) (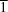
10) and (002); for (Ie) (002), (111) and (012); for (IIa) (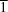
11); and for (IId) (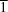
12). In addition, the bad outlier reflections (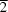
04) for (Id) and (130) for (IIe) were also omitted. All H atoms were located in difference maps and then treated as riding atoms in geometrically idealized positions with C—H distances 0.93 Å (aromatic), 0.96 Å (CH_3_) or 0.97 Å (CH_2_), and with *U*_iso_(H) = *kU*_eq_(C), where *k* = 1.5 for the methyl groups which were permitted to rotate but not to tilt, and 1.2 for all other H atoms. The final difference map for compound (Ie) contained two significant peaks, 0.85 e Å^−3^ at (0.227, 0.557, 0.598), and 0.81 e Å^−3^ at (0.380, 0.488, 0.596), respectively 1.27 and 1.14 Å from atom C351: however, attempts to develop a plausible disorder model based upon these two peaks were not fruitful. For the minor disorder compound in compound (IId), the bonded distances and the [1,3] non-bonded distances were restrained to be the same as the corresponding distances in the major disorder component, subject to s.u. values of 0.01 and 0.02 Å, respectively. In addition, similarity restraints were applied to the anisotropic displacement parameters of the partial-occupancy atoms in the disorder components. Subject to these conditions, the occupancies for the two disorder components refined to values of 0.55 (4) and 0.45 (4). Examination of the final refined structures using *PLATON* (Spek, 2020 ▸) showed that the structure of compound (IIa) contained a void space, of volume 64 Å^3^, centred at (0.5, 0, 0), but further examination of this structure using the SQUEEZE procedure (Spek, 2015 ▸) showed that the void contained negligible electron density, consistent with the final difference map.

## Supplementary Material

Crystal structure: contains datablock(s) global, Ib, Ic, Ie, IIa, IId, IIe. DOI: 10.1107/S2056989020005113/mw2158sup1.cif

Structure factors: contains datablock(s) Ib. DOI: 10.1107/S2056989020005113/mw2158Ibsup2.hkl

Supporting information file. DOI: 10.1107/S2056989020005113/mw2158Ibsup8.cml

Structure factors: contains datablock(s) Ic. DOI: 10.1107/S2056989020005113/mw2158Icsup3.hkl

Supporting information file. DOI: 10.1107/S2056989020005113/mw2158Icsup9.cml

Structure factors: contains datablock(s) Ie. DOI: 10.1107/S2056989020005113/mw2158Iesup4.hkl

Supporting information file. DOI: 10.1107/S2056989020005113/mw2158IIasup10.cml

Structure factors: contains datablock(s) IIa. DOI: 10.1107/S2056989020005113/mw2158IIasup5.hkl

Supporting information file. DOI: 10.1107/S2056989020005113/mw2158IIdsup11.cml

Structure factors: contains datablock(s) IId. DOI: 10.1107/S2056989020005113/mw2158IIdsup6.hkl

Structure factors: contains datablock(s) IIe. DOI: 10.1107/S2056989020005113/mw2158IIesup7.hkl

CCDC references: 1996407, 1996406, 1996405, 1996404, 1996403, 1996402

Additional supporting information:  crystallographic information; 3D view; checkCIF report

## Figures and Tables

**Figure 2:**
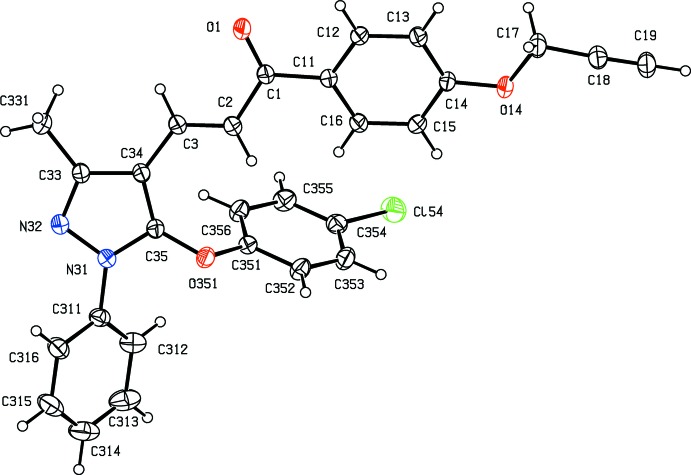
The mol­ecular structure of compound (Ic) showing the atom-labelling scheme. Displacement ellipsoids are drawn at the 30% probability level.

**Figure 4:**
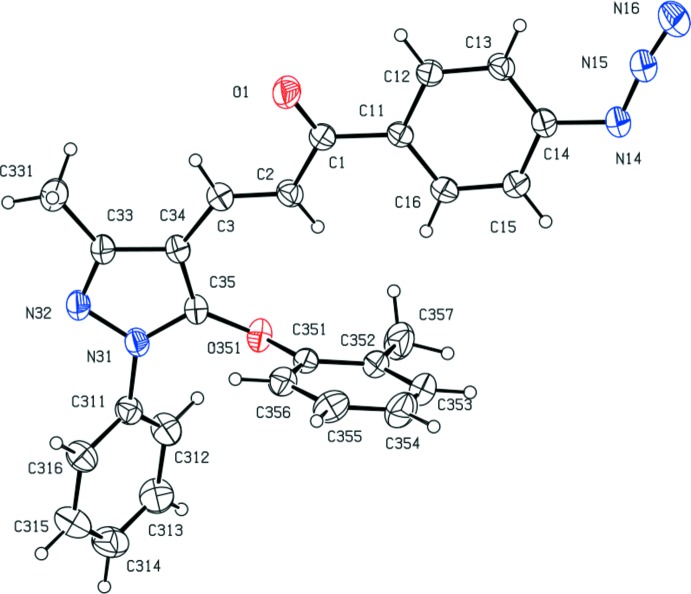
The mol­ecular structure of compound (IIa) showing the atom-labelling scheme. Displacement ellipsoids are drawn at the 30% probability level.

**Figure 6:**
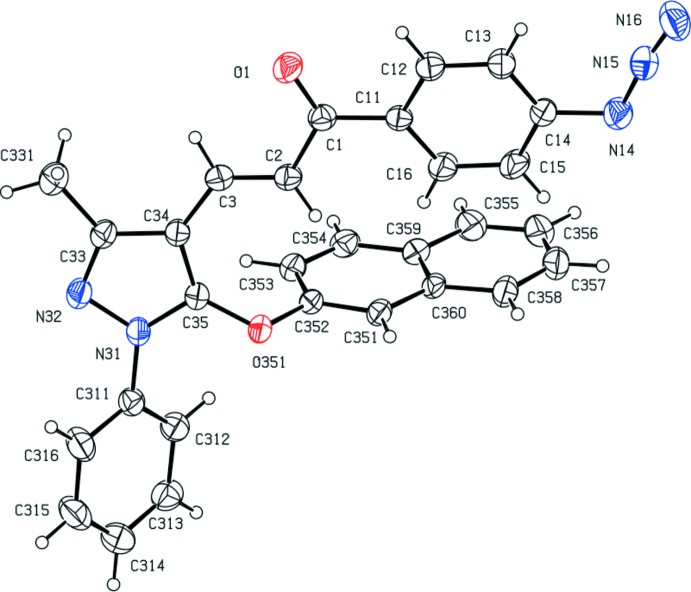
The mol­ecular structure of compound (IIe) showing the atom-labelling scheme. Displacement ellipsoids are drawn at the 30% probability level.

**Table 1:** Selected torsional angles (°) for compounds (Ib), (Ic), (Ie), (IIa), (IId) and (IIe)

Parameter	(Ib)	(Ic)	(Ie)	(IIa)	(IId)	(IIe)
N32—N31—C311—C312	151.1 (3)	137.0 (2)	139.9 (3)	135.1 (2)	149.6 (4)	140.9 (3)
C2—C1—C11—C12	168.8 (2)	−163.4 (2)	−162.8 (3)	166.8 (2)	−172.7 (4)	−171.4 (3)
C13—C14—O14—C17	169.8 (2)	3.5 (3)	−0.4 (4)			
C14—O14—C17—C18	−68.7 (3)	−177.1 (2)	−174.7 (3)			
C13—C14—N14—N15				−2.7 (3)	−172.6 (5)	−5.0 (6)
C34—C35—O351—C351	−76.7 (3)	−69.3 (3)		70.1 (3)	78.3 (16)	
C34—C35—O451—C451					65.0 (12)	
C35—O351—C351—C352	157.6 (2)	169.5 (2)		−159.6 (2)	−164 (2)	
C35—O451—C451—C45					−163.6 (18)	
C34—C35—O351—C352			−70.6 (4)			−71.1 (5)
C35—O351—C352—C351			161.8 (2)			150.9 (3)

**Table 2:** Hydrogen bonds and short intra- and inter-mol­ecular contacts (Å, °) for compounds (Ib), (Ic), (Ie), (IIa), (IId) and (IIe) *Cg*1, *Cg*2, *Cg*3 and *Cg*4 represent the centroids of the rings C311–C316), (C351–C356), (C351–C354/C359/C360) and (C355–C360), respectively

Compound	*D*—H⋯*A*	*D*—*A*	H⋯*A*	*D*⋯*A*	*D*—H⋯*A*
(Ib)	C355—H355⋯O14^i^	0.93	2.59	3.468 (4)	158
	C356—H356⋯O1^ii^	0.93	2.51	3.360 (4)	152
(Ic)	C19—H19⋯O1^iii^	0.93	2.25	3.161 (3)	165
	C16—H16⋯*Cg*2	0.93	2.98	3.882 (2)	165
	C356—H356⋯*Cg*1^iv^	0.93	2.88	3.685 (2)	146
(Ie)	C19—H19⋯O1^v^	0.93	2.32	3.233 (5)	165
	C353—H353⋯*Cg*1^vi^	0.93	2.86	3.708 (3)	152
(IIa)	C357—H35*B*⋯O1^vii^	0.96	2.51	3.396 (4)	154
(IId)	C356—H356⋯O1^viii^	0.93	2.32	3.115 (18)	143
	C456—H456⋯O1^viii^	0.93	2.47	3.21 (2)	137
(IIe)	C353—H353⋯O1^ix^	0.93	2.47	3.288 (4)	147
	C12—H12⋯*Cg*3^*x*^	0.93	2.93	3.761 (4)	150
	C13—H13⋯*Cg*4^*x*^	0.93	2.73	3.547 (4)	148

Symmmetry codes: (i) 2 − *x*, −*y*, 1 − *z*; (ii) 2 − *x*, 1 − *y*, 1 − *z*; (iii) 1 + *x*, 1 + *y*, *z*; (iv) 1 − *x*, −*y*, 2 − *z*; (v) −1 + *x*, 1 + *y*, *z*; (vi) 1 − *x*, −*y*, 1 − *z*; (vii) 1 − *x*, 1 − *y*, 2 − *z*; (viii) 1 − *x*, 2 − *y*, 1 − *z*; (ix) 1 − *x*, 1 − *y*, −*z*; (*x*) −1 + *x*, *y*, *z*.

**Table:** 

	(Ib)	(Ic)	(Ie)
Crystal data			
Chemical formula	C_28_H_21_ClN_2_O_3_	C_28_H_21_ClN_2_O_3_	C_32_H_24_N_2_O_3_
*M*_r_	468.92	468.92	484.53
Crystal system, space group	Triclinic, *P* 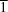	Triclinic, *P* 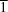	Triclinic, *P* 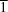
Temperature (K)	297	297	297
*a*, *b*, *c* (Å)	9.909 (7), 10.193 (6), 12.024 (8)	8.9959 (14), 9.7380 (15), 13.637 (2)	8.8615 (6), 10.4973 (7), 13.6588 (10)
α, β, γ (°)	90.94 (2), 106.27 (2), 92.75 (2)	95.901 (4), 94.122 (4), 95.959 (4)	79.006 (3), 89.412 (3), 80.971 (3)
*V* (Å^3^)	1163.9 (13)	1177.8 (3)	1231.54 (15)
*Z*	2	2	2
Radiation type	Mo *K*α	Mo *K*α	Mo *K*α
μ (mm^−1^)	0.20	0.20	0.08
Crystal size (mm)	0.18 × 0.15 × 0.10	0.20 × 0.15 × 0.15	0.20 × 0.16 × 0.16

Data collection			
Diffractometer	Bruker APEXII	Bruker APEXII	Bruker APEXII
Absorption correction	Multi-scan (*SADABS*; Bruker, 2016 ▸)	Multi-scan (*SADABS*; Bruker, 2016 ▸)	Multi-scan (*SADABS*; Bruker, 2016 ▸)
*T*_min_, *T*_max_	0.833, 0.980	0.901, 0.971	0.898, 0.987
No. of measured, independent and observed [*I* > 2σ(*I*)] reflections	43789, 5884, 4402	49011, 4837, 3930	30613, 4373, 3474
*R*_int_	0.061	0.061	0.055
(sin θ/λ)_max_ (Å^−1^)	0.672	0.629	0.598

Refinement			
*R*[*F* ^2^ > 2σ(*F* ^2^)], *wR*(*F* ^2^), *S*	0.058, 0.155, 1.10	0.048, 0.125, 1.15	0.070, 0.187, 1.07
No. of reflections	5884	4837	4373
No. of parameters	309	309	336
No. of restraints	0	0	0
H-atom treatment	H-atom parameters constrained	H-atom parameters constrained	H-atom parameters constrained
Δρ_max_, Δρ_min_ (e Å^−3^)	0.43, −0.41	0.35, −0.45	0.85, −0.29

**Table:** 

	(IIa)	(IId)	(IIe)
Crystal data			
Chemical formula	C_26_H_21_N_5_O_2_	C_25_H_17_Cl_2_N_5_O_2_	C_29_H_21_N_5_O_2_
*M*_r_	435.48	490.33	471.51
Crystal system, space group	Triclinic, *P* 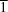	Monoclinic, *C*2/*c*	Monoclinic, *P*2_1_/*n*
Temperature (K)	297	297	297
*a*, *b*, *c* (Å)	9.8432 (6), 11.7441 (7), 12.3005 (7)	28.1916 (17), 8.0537 (5), 22.0446 (12)	9.8460 (8), 22.4303 (18), 11.0490 (9)
α, β, γ (°)	114.120 (2), 111.139 (2), 96.537 (2)	90, 109.070 (1), 90	90, 104.157 (2), 90
*V* (Å^3^)	1152.06 (12)	4730.5 (5)	2366.0 (3)
*Z*	2	8	4
Radiation type	Mo *K*α	Mo *K*α	Mo *K*α
μ (mm^−1^)	0.08	0.31	0.09
Crystal size (mm)	0.20 × 0.20 × 0.18	0.18 × 0.15 × 0.15	0.22 × 0.21 × 0.16

Data collection			
Diffractometer	Bruker APEXII	Bruker APEXII	Bruker APEXII
Absorption correction	Multi-scan (*SADABS*; Bruker, 2016 ▸)	Multi-scan (*SADABS*; Bruker, 2016 ▸)	Multi-scan (*SADABS*; Bruker, 2016 ▸)
*T*_min_, *T*_max_	0.868, 0.985	0.881, 0.955	0.930, 0.986
No. of measured, independent and observed [*I* > 2σ(*I*)] reflections	17379, 4050, 2957	31508, 4174, 3181	43217, 4196, 2463
*R*_int_	0.048	0.049	0.092
(sin θ/λ)_max_ (Å^−1^)	0.596	0.595	0.597

Refinement			
*R*[*F* ^2^ > 2σ(*F* ^2^)], *wR*(*F* ^2^), *S*	0.050, 0.148, 1.10	0.086, 0.155, 1.36	0.063, 0.154, 1.06
No. of reflections	4050	4174	4196
No. of parameters	301	382	327
No. of restraints	0	291	0
H-atom treatment	H-atom parameters constrained	H-atom parameters constrained	H-atom parameters constrained
Δρ_max_, Δρ_min_ (e Å^−3^)	0.19, −0.22	0.21, −0.23	0.23, −0.27

Computer programs: *APEX3*, *SAINT* and *XPREP* (Bruker, 2016 ▸), *SHELXT2014/5* (Sheldrick, 2015*a* ▸), *SHELXL2014* (Sheldrick, 2015*b* ▸) and *PLATON* (Spek, 2020 ▸).

## supplementary crystallographic information

### 3-[5-(2-Chlorophenoxy)-3-methyl-1-phenyl-1H-pyrazol-4-yl]-1-[4-(prop-2-yn-1-yloxy)phenyl]prop-2-en-1-one (Ib) Crystal data

**Table:**

C_28_H_21_ClN_2_O_3_	*Z* = 2
*M**_r_* = 468.92	*F*(000) = 488
Triclinic, *P*1	*D*_x_ = 1.338 Mg m^−^^3^
*a* = 9.909 (7) Å	Mo *K*α radiation, λ = 0.71073 Å
*b* = 10.193 (6) Å	Cell parameters from 7088 reflections
*c* = 12.024 (8) Å	θ = 3.0–31.0°
α = 90.94 (2)°	µ = 0.20 mm^−^^1^
β = 106.27 (2)°	*T* = 297 K
γ = 92.75 (2)°	Block, colourless
*V* = 1163.9 (13) Å^3^	0.18 × 0.15 × 0.10 mm

### 3-[5-(2-Chlorophenoxy)-3-methyl-1-phenyl-1H-pyrazol-4-yl]-1-[4-(prop-2-yn-1-yloxy)phenyl]prop-2-en-1-one (Ib) Data collection

**Table:**

Bruker APEXII diffractometer	5884 independent reflections
Radiation source: fine focussealed tube	4402 reflections with *I* > 2σ(*I*)
Graphite monochromator	*R*_int_ = 0.061
φ and ω scans	θ_max_ = 28.6°, θ_min_ = 3.6°
Absorption correction: multi-scan (SADABS; Bruker, 2016)	*h* = −13→13
*T*_min_ = 0.833, *T*_max_ = 0.980	*k* = −13→13
43789 measured reflections	*l* = −16→16

### 3-[5-(2-Chlorophenoxy)-3-methyl-1-phenyl-1H-pyrazol-4-yl]-1-[4-(prop-2-yn-1-yloxy)phenyl]prop-2-en-1-one (Ib) Refinement

**Table:**

Refinement on *F*^2^	Hydrogen site location: inferred from neighbouring sites
Least-squares matrix: full	H-atom parameters constrained
*R*[*F*^2^ > 2σ(*F*^2^)] = 0.058	*w* = 1/[σ^2^(*F*_o_^2^) + (0.0448*P*)^2^ + 0.9732*P*] where *P* = (*F*_o_^2^ + 2*F*_c_^2^)/3
*wR*(*F*^2^) = 0.155	(Δ/σ)_max_ < 0.001
*S* = 1.10	Δρ_max_ = 0.43 e Å^−^^3^
5884 reflections	Δρ_min_ = −0.41 e Å^−^^3^
309 parameters	Extinction correction: SHELXL, Fc^*^=kFc[1+0.001xFc^2^λ^3^/sin(2θ)]^-1/4^
0 restraints	Extinction coefficient: 0.154 (8)
Primary atom site location: dual	

### 3-[5-(2-Chlorophenoxy)-3-methyl-1-phenyl-1H-pyrazol-4-yl]-1-[4-(prop-2-yn-1-yloxy)phenyl]prop-2-en-1-one (Ib) Special details

**Table:**

Geometry. All esds (except the esd in the dihedral angle between two l.s. planes) are estimated using the full covariance matrix. The cell esds are taken into account individually in the estimation of esds in distances, angles and torsion angles; correlations between esds in cell parameters are only used when they are defined by crystal symmetry. An approximate (isotropic) treatment of cell esds is used for estimating esds involving l.s. planes.

### 3-[5-(2-Chlorophenoxy)-3-methyl-1-phenyl-1H-pyrazol-4-yl]-1-[4-(prop-2-yn-1-yloxy)phenyl]prop-2-en-1-one (Ib) Fractional atomic coordinates and isotropic or equivalent isotropic displacement parameters (Å^2^)

**Table:**

	*x*	*y*	*z*	*U*_iso_*/*U*_eq_	
C1	0.9482 (2)	0.3833 (2)	0.72250 (19)	0.0464 (5)	
O1	1.0416 (2)	0.45886 (19)	0.78220 (17)	0.0709 (6)	
C2	0.8469 (2)	0.4294 (2)	0.6173 (2)	0.0478 (5)	
H2	0.7655	0.3779	0.5825	0.057*	
C3	0.8698 (2)	0.5434 (2)	0.57132 (18)	0.0405 (4)	
H3	0.9508	0.5925	0.6116	0.049*	
C11	0.9385 (2)	0.2433 (2)	0.75289 (18)	0.0417 (5)	
C12	1.0483 (2)	0.1947 (2)	0.8398 (2)	0.0486 (5)	
H12	1.1247	0.2507	0.8778	0.058*	
C13	1.0451 (2)	0.0655 (2)	0.8700 (2)	0.0488 (5)	
H13	1.1197	0.0347	0.9274	0.059*	
C14	0.9314 (2)	−0.0192 (2)	0.8155 (2)	0.0446 (5)	
C15	0.8204 (3)	0.0268 (2)	0.7302 (2)	0.0511 (6)	
H15	0.7431	−0.0292	0.6939	0.061*	
C16	0.8253 (2)	0.1567 (2)	0.6991 (2)	0.0496 (5)	
H16	0.7511	0.1868	0.6410	0.060*	
O14	0.94132 (19)	−0.14583 (16)	0.85166 (17)	0.0576 (5)	
C17	0.8187 (3)	−0.2343 (3)	0.8139 (3)	0.0654 (7)	
H17A	0.8452	−0.3230	0.8345	0.078*	
H17B	0.7829	−0.2328	0.7301	0.078*	
C18	0.7077 (3)	−0.2002 (3)	0.8653 (3)	0.0608 (7)	
C19	0.6186 (4)	−0.1767 (4)	0.9061 (3)	0.0824 (10)	
H19	0.5469	−0.1578	0.9390	0.099*	
N31	0.62180 (18)	0.64397 (15)	0.30547 (16)	0.0394 (4)	
N32	0.71026 (19)	0.75523 (16)	0.33267 (16)	0.0425 (4)	
C33	0.8087 (2)	0.72855 (19)	0.42829 (19)	0.0394 (4)	
C34	0.7862 (2)	0.60108 (18)	0.46748 (18)	0.0373 (4)	
C35	0.6653 (2)	0.55241 (18)	0.38607 (18)	0.0368 (4)	
C311	0.4990 (2)	0.6447 (2)	0.20822 (19)	0.0424 (5)	
C312	0.4418 (4)	0.5309 (3)	0.1454 (3)	0.0775 (10)	
H312	0.4841	0.4517	0.1647	0.093*	
C313	0.3214 (4)	0.5351 (3)	0.0534 (3)	0.0871 (11)	
H313	0.2826	0.4581	0.0117	0.104*	
C314	0.2589 (3)	0.6505 (3)	0.0231 (2)	0.0684 (8)	
H314	0.1766	0.6523	−0.0375	0.082*	
C315	0.3188 (4)	0.7634 (3)	0.0829 (3)	0.0777 (9)	
H315	0.2781	0.8428	0.0615	0.093*	
C316	0.4393 (3)	0.7612 (3)	0.1752 (2)	0.0639 (7)	
H316	0.4797	0.8390	0.2146	0.077*	
C331	0.9235 (3)	0.8287 (2)	0.4843 (2)	0.0514 (6)	
H33A	0.9224	0.9001	0.4330	0.077*	
H33B	1.0128	0.7893	0.5005	0.077*	
H33C	0.9093	0.8613	0.5553	0.077*	
O351	0.58686 (15)	0.43808 (13)	0.38387 (13)	0.0395 (3)	
C351	0.6396 (2)	0.32381 (17)	0.35285 (17)	0.0335 (4)	
C352	0.5909 (2)	0.20629 (18)	0.38930 (17)	0.0358 (4)	
Cl52	0.47497 (6)	0.21019 (6)	0.47407 (5)	0.05086 (19)	
C353	0.6332 (2)	0.08826 (19)	0.35823 (19)	0.0431 (5)	
H353	0.6008	0.0102	0.3832	0.052*	
C354	0.7248 (3)	0.0858 (2)	0.2892 (2)	0.0478 (5)	
H354	0.7528	0.0060	0.2671	0.057*	
C355	0.7740 (2)	0.2023 (2)	0.2537 (2)	0.0455 (5)	
H355	0.8358	0.2006	0.2081	0.055*	
C356	0.7321 (2)	0.32177 (19)	0.28541 (18)	0.0396 (4)	
H356	0.7659	0.3999	0.2616	0.048*	

### 3-[5-(2-Chlorophenoxy)-3-methyl-1-phenyl-1H-pyrazol-4-yl]-1-[4-(prop-2-yn-1-yloxy)phenyl]prop-2-en-1-one (Ib) Atomic displacement parameters (Å^2^)

**Table:**

	*U*^11^	*U*^22^	*U*^33^	*U*^12^	*U*^13^	*U*^23^
C1	0.0496 (12)	0.0438 (12)	0.0403 (11)	−0.0114 (9)	0.0060 (9)	0.0047 (9)
O1	0.0757 (13)	0.0561 (11)	0.0594 (11)	−0.0278 (9)	−0.0116 (9)	0.0101 (9)
C2	0.0484 (12)	0.0413 (11)	0.0463 (12)	−0.0082 (9)	0.0027 (10)	0.0037 (9)
C3	0.0404 (10)	0.0360 (10)	0.0430 (11)	−0.0028 (8)	0.0092 (8)	−0.0004 (8)
C11	0.0438 (11)	0.0435 (11)	0.0346 (10)	−0.0065 (9)	0.0072 (8)	0.0038 (8)
C12	0.0434 (11)	0.0512 (13)	0.0443 (11)	−0.0071 (9)	0.0027 (9)	0.0045 (10)
C13	0.0434 (12)	0.0514 (13)	0.0478 (12)	0.0026 (10)	0.0060 (9)	0.0089 (10)
C14	0.0501 (12)	0.0399 (11)	0.0460 (11)	0.0008 (9)	0.0174 (10)	0.0028 (9)
C15	0.0495 (13)	0.0445 (12)	0.0525 (13)	−0.0089 (10)	0.0050 (10)	0.0025 (10)
C16	0.0462 (12)	0.0477 (12)	0.0466 (12)	−0.0074 (10)	0.0005 (10)	0.0091 (10)
O14	0.0601 (10)	0.0410 (9)	0.0728 (12)	0.0041 (7)	0.0196 (9)	0.0106 (8)
C17	0.0797 (19)	0.0404 (13)	0.0728 (18)	−0.0059 (12)	0.0178 (15)	0.0014 (12)
C18	0.0536 (15)	0.0500 (14)	0.0678 (17)	−0.0125 (11)	0.0010 (13)	0.0083 (12)
C19	0.0546 (17)	0.088 (2)	0.096 (2)	−0.0109 (16)	0.0100 (17)	0.0045 (19)
N31	0.0418 (9)	0.0256 (8)	0.0468 (9)	−0.0026 (6)	0.0067 (7)	0.0027 (7)
N32	0.0465 (10)	0.0249 (8)	0.0515 (10)	−0.0059 (7)	0.0079 (8)	0.0017 (7)
C33	0.0426 (11)	0.0283 (9)	0.0468 (11)	−0.0032 (8)	0.0127 (9)	−0.0023 (8)
C34	0.0393 (10)	0.0273 (9)	0.0443 (11)	−0.0002 (7)	0.0104 (8)	0.0000 (8)
C35	0.0401 (10)	0.0243 (8)	0.0460 (11)	−0.0019 (7)	0.0127 (8)	0.0007 (7)
C311	0.0429 (11)	0.0378 (10)	0.0443 (11)	−0.0002 (8)	0.0085 (9)	0.0063 (8)
C312	0.086 (2)	0.0420 (14)	0.0768 (19)	−0.0110 (13)	−0.0211 (16)	0.0075 (13)
C313	0.094 (2)	0.0627 (18)	0.074 (2)	−0.0247 (16)	−0.0216 (18)	0.0134 (15)
C314	0.0563 (15)	0.089 (2)	0.0507 (14)	−0.0010 (14)	0.0002 (12)	0.0174 (14)
C315	0.087 (2)	0.076 (2)	0.0599 (17)	0.0339 (17)	−0.0013 (15)	0.0082 (15)
C316	0.0765 (18)	0.0487 (14)	0.0565 (15)	0.0156 (13)	0.0005 (13)	−0.0013 (11)
C331	0.0540 (13)	0.0357 (11)	0.0585 (14)	−0.0118 (9)	0.0084 (11)	−0.0002 (10)
O351	0.0391 (7)	0.0245 (6)	0.0563 (9)	−0.0031 (5)	0.0164 (6)	0.0009 (6)
C351	0.0338 (9)	0.0260 (8)	0.0374 (9)	−0.0023 (7)	0.0055 (7)	0.0005 (7)
C352	0.0353 (9)	0.0307 (9)	0.0382 (10)	−0.0063 (7)	0.0063 (8)	0.0020 (7)
Cl52	0.0522 (3)	0.0465 (3)	0.0582 (4)	−0.0102 (2)	0.0248 (3)	0.0016 (2)
C353	0.0521 (12)	0.0265 (9)	0.0479 (11)	−0.0043 (8)	0.0106 (9)	0.0040 (8)
C354	0.0604 (14)	0.0322 (10)	0.0508 (12)	0.0068 (9)	0.0149 (11)	0.0003 (9)
C355	0.0481 (12)	0.0432 (11)	0.0486 (12)	0.0061 (9)	0.0187 (10)	0.0035 (9)
C356	0.0409 (10)	0.0322 (10)	0.0460 (11)	−0.0023 (8)	0.0129 (9)	0.0062 (8)

### 3-[5-(2-Chlorophenoxy)-3-methyl-1-phenyl-1H-pyrazol-4-yl]-1-[4-(prop-2-yn-1-yloxy)phenyl]prop-2-en-1-one (Ib) Geometric parameters (Å, º)

**Table:**

C1—O1	1.228 (3)	C33—C331	1.492 (3)
C1—C2	1.477 (3)	C34—C35	1.382 (3)
C1—C11	1.484 (3)	C35—O351	1.365 (2)
C2—C3	1.331 (3)	C311—C316	1.367 (3)
C2—H2	0.9300	C311—C312	1.380 (3)
C3—C34	1.443 (3)	C312—C313	1.384 (4)
C3—H3	0.9300	C312—H312	0.9300
C11—C16	1.391 (3)	C313—C314	1.363 (5)
C11—C12	1.398 (3)	C313—H313	0.9300
C12—C13	1.373 (3)	C314—C315	1.364 (5)
C12—H12	0.9300	C314—H314	0.9300
C13—C14	1.385 (3)	C315—C316	1.385 (4)
C13—H13	0.9300	C315—H315	0.9300
C14—O14	1.368 (3)	C316—H316	0.9300
C14—C15	1.384 (3)	C331—H33A	0.9600
C15—C16	1.384 (3)	C331—H33B	0.9600
C15—H15	0.9300	C331—H33C	0.9600
C16—H16	0.9300	O351—C351	1.387 (2)
O14—C17	1.438 (3)	C351—C356	1.384 (3)
C17—C18	1.458 (4)	C351—C352	1.393 (3)
C17—H17A	0.9700	C352—C353	1.373 (3)
C17—H17B	0.9700	C352—Cl52	1.737 (2)
C18—C19	1.157 (5)	C353—C354	1.391 (3)
C19—H19	0.9300	C353—H353	0.9300
N31—C35	1.353 (3)	C354—C355	1.382 (3)
N31—N32	1.376 (2)	C354—H354	0.9300
N31—C311	1.433 (3)	C355—C356	1.386 (3)
N32—C33	1.325 (3)	C355—H355	0.9300
C33—C34	1.418 (3)	C356—H356	0.9300
			
O1—C1—C2	120.4 (2)	N31—C35—O351	120.93 (18)
O1—C1—C11	120.4 (2)	N31—C35—C34	109.00 (17)
C2—C1—C11	119.14 (19)	O351—C35—C34	129.85 (19)
C3—C2—C1	121.3 (2)	C316—C311—C312	119.3 (2)
C3—C2—H2	119.3	C316—C311—N31	119.2 (2)
C1—C2—H2	119.3	C312—C311—N31	121.5 (2)
C2—C3—C34	128.8 (2)	C311—C312—C313	119.8 (3)
C2—C3—H3	115.6	C311—C312—H312	120.1
C34—C3—H3	115.6	C313—C312—H312	120.1
C16—C11—C12	117.7 (2)	C314—C313—C312	120.9 (3)
C16—C11—C1	123.4 (2)	C314—C313—H313	119.6
C12—C11—C1	118.86 (19)	C312—C313—H313	119.6
C13—C12—C11	121.1 (2)	C313—C314—C315	119.1 (3)
C13—C12—H12	119.5	C313—C314—H314	120.5
C11—C12—H12	119.5	C315—C314—H314	120.5
C12—C13—C14	120.4 (2)	C314—C315—C316	120.9 (3)
C12—C13—H13	119.8	C314—C315—H315	119.6
C14—C13—H13	119.8	C316—C315—H315	119.6
O14—C14—C15	125.2 (2)	C311—C316—C315	120.0 (3)
O14—C14—C13	115.0 (2)	C311—C316—H316	120.0
C15—C14—C13	119.8 (2)	C315—C316—H316	120.0
C16—C15—C14	119.5 (2)	C33—C331—H33A	109.5
C16—C15—H15	120.2	C33—C331—H33B	109.5
C14—C15—H15	120.2	H33A—C331—H33B	109.5
C15—C16—C11	121.6 (2)	C33—C331—H33C	109.5
C15—C16—H16	119.2	H33A—C331—H33C	109.5
C11—C16—H16	119.2	H33B—C331—H33C	109.5
C14—O14—C17	118.3 (2)	C35—O351—C351	117.09 (16)
O14—C17—C18	112.3 (2)	C356—C351—O351	123.40 (17)
O14—C17—H17A	109.1	C356—C351—C352	119.82 (18)
C18—C17—H17A	109.1	O351—C351—C352	116.73 (18)
O14—C17—H17B	109.1	C353—C352—C351	120.49 (19)
C18—C17—H17B	109.1	C353—C352—Cl52	120.13 (15)
H17A—C17—H17B	107.9	C351—C352—Cl52	119.38 (16)
C19—C18—C17	178.1 (3)	C352—C353—C354	119.80 (19)
C18—C19—H19	180.0	C352—C353—H353	120.1
C35—N31—N32	110.06 (17)	C354—C353—H353	120.1
C35—N31—C311	130.75 (17)	C355—C354—C353	119.8 (2)
N32—N31—C311	118.98 (16)	C355—C354—H354	120.1
C33—N32—N31	105.58 (16)	C353—C354—H354	120.1
N32—C33—C34	112.02 (18)	C354—C355—C356	120.6 (2)
N32—C33—C331	120.60 (19)	C354—C355—H355	119.7
C34—C33—C331	127.3 (2)	C356—C355—H355	119.7
C35—C34—C33	103.31 (18)	C351—C356—C355	119.51 (18)
C35—C34—C3	130.32 (19)	C351—C356—H356	120.2
C33—C34—C3	126.33 (19)	C355—C356—H356	120.2
			
O1—C1—C2—C3	13.8 (4)	C311—N31—C35—C34	−175.8 (2)
C11—C1—C2—C3	−164.2 (2)	C33—C34—C35—N31	0.5 (2)
C1—C2—C3—C34	177.4 (2)	C3—C34—C35—N31	178.0 (2)
O1—C1—C11—C16	171.0 (2)	C33—C34—C35—O351	−174.0 (2)
C2—C1—C11—C16	−11.1 (4)	C3—C34—C35—O351	3.5 (4)
O1—C1—C11—C12	−9.2 (4)	C35—N31—C311—C316	147.1 (3)
C2—C1—C11—C12	168.8 (2)	N32—N31—C311—C316	−27.1 (3)
C16—C11—C12—C13	0.7 (4)	C35—N31—C311—C312	−34.7 (4)
C1—C11—C12—C13	−179.2 (2)	N32—N31—C311—C312	151.1 (3)
C11—C12—C13—C14	−0.8 (4)	C316—C311—C312—C313	−3.1 (5)
C12—C13—C14—O14	179.0 (2)	N31—C311—C312—C313	178.7 (3)
C12—C13—C14—C15	−0.1 (4)	C311—C312—C313—C314	0.7 (6)
O14—C14—C15—C16	−178.1 (2)	C312—C313—C314—C315	1.7 (6)
C13—C14—C15—C16	0.9 (4)	C313—C314—C315—C316	−1.6 (5)
C14—C15—C16—C11	−1.0 (4)	C312—C311—C316—C315	3.1 (5)
C12—C11—C16—C15	0.1 (4)	N31—C311—C316—C315	−178.6 (3)
C1—C11—C16—C15	−180.0 (2)	C314—C315—C316—C311	−0.8 (5)
C15—C14—O14—C17	−11.2 (4)	N31—C35—O351—C351	109.4 (2)
C13—C14—O14—C17	169.8 (2)	C34—C35—O351—C351	−76.7 (3)
C14—O14—C17—C18	−68.7 (3)	C35—O351—C351—C356	−24.8 (3)
C35—N31—N32—C33	1.5 (2)	C35—O351—C351—C352	157.64 (18)
C311—N31—N32—C33	176.81 (18)	C356—C351—C352—C353	−0.4 (3)
N31—N32—C33—C34	−1.2 (2)	O351—C351—C352—C353	177.27 (18)
N31—N32—C33—C331	−179.43 (19)	C356—C351—C352—Cl52	179.92 (15)
N32—C33—C34—C35	0.5 (2)	O351—C351—C352—Cl52	−2.4 (2)
C331—C33—C34—C35	178.5 (2)	C351—C352—C353—C354	−0.4 (3)
N32—C33—C34—C3	−177.2 (2)	Cl52—C352—C353—C354	179.26 (17)
C331—C33—C34—C3	0.9 (4)	C352—C353—C354—C355	0.9 (3)
C2—C3—C34—C35	−3.3 (4)	C353—C354—C355—C356	−0.5 (4)
C2—C3—C34—C33	173.7 (2)	O351—C351—C356—C355	−176.76 (19)
N32—N31—C35—O351	173.80 (17)	C352—C351—C356—C355	0.8 (3)
C311—N31—C35—O351	−0.8 (3)	C354—C355—C356—C351	−0.3 (3)
N32—N31—C35—C34	−1.3 (2)		

### 3-[5-(2-Chlorophenoxy)-3-methyl-1-phenyl-1H-pyrazol-4-yl]-1-[4-(prop-2-yn-1-yloxy)phenyl]prop-2-en-1-one (Ib) Hydrogen-bond geometry (Å, º)

**Table:**

*D*—H···*A*	*D*—H	H···*A*	*D*···*A*	*D*—H···*A*
C355—H355···O14^i^	0.93	2.59	3.468 (4)	158
C356—H356···O1^ii^	0.93	2.51	3.360 (4)	152

Symmetry codes: (i) −*x*+2, −*y*, −*z*+1; (ii) −*x*+2, −*y*+1, −*z*+1.

### 3-[5-(4-Chlorophenoxy)-3-methyl-1-phenyl-1H-pyrazol-4-yl]-1-[4-(prop-2-yn-1-yloxy)phenyl]prop-2-en-1-one (Ic) Crystal data

**Table:**

C_28_H_21_ClN_2_O_3_	*Z* = 2
*M**_r_* = 468.92	*F*(000) = 488
Triclinic, *P*1	*D*_x_ = 1.322 Mg m^−^^3^
*a* = 8.9959 (14) Å	Mo *K*α radiation, λ = 0.71073 Å
*b* = 9.7380 (15) Å	Cell parameters from 4839 reflections
*c* = 13.637 (2) Å	θ = 2.9–26.5°
α = 95.901 (4)°	µ = 0.20 mm^−^^1^
β = 94.122 (4)°	*T* = 297 K
γ = 95.959 (4)°	Block, brown
*V* = 1177.8 (3) Å^3^	0.20 × 0.15 × 0.15 mm

### 3-[5-(4-Chlorophenoxy)-3-methyl-1-phenyl-1H-pyrazol-4-yl]-1-[4-(prop-2-yn-1-yloxy)phenyl]prop-2-en-1-one (Ic) Data collection

**Table:**

Bruker APEXII diffractometer	4837 independent reflections
Radiation source: fine focussealed tube	3930 reflections with *I* > 2σ(*I*)
Graphite monochromator	*R*_int_ = 0.061
φ and ω scans	θ_max_ = 26.5°, θ_min_ = 3.3°
Absorption correction: multi-scan (SADABS; Bruker, 2016)	*h* = −11→11
*T*_min_ = 0.901, *T*_max_ = 0.971	*k* = −12→12
49011 measured reflections	*l* = −17→17

### 3-[5-(4-Chlorophenoxy)-3-methyl-1-phenyl-1H-pyrazol-4-yl]-1-[4-(prop-2-yn-1-yloxy)phenyl]prop-2-en-1-one (Ic) Refinement

**Table:**

Refinement on *F*^2^	Hydrogen site location: inferred from neighbouring sites
Least-squares matrix: full	H-atom parameters constrained
*R*[*F*^2^ > 2σ(*F*^2^)] = 0.048	*w* = 1/[σ^2^(*F*_o_^2^) + (0.0384*P*)^2^ + 0.6832*P*] where *P* = (*F*_o_^2^ + 2*F*_c_^2^)/3
*wR*(*F*^2^) = 0.125	(Δ/σ)_max_ = 0.001
*S* = 1.15	Δρ_max_ = 0.35 e Å^−^^3^
4837 reflections	Δρ_min_ = −0.45 e Å^−^^3^
309 parameters	Extinction correction: SHELXL, Fc^*^=kFc[1+0.001xFc^2^λ^3^/sin(2θ)]^-1/4^
0 restraints	Extinction coefficient: 0.092 (6)
Primary atom site location: dual	

### 3-[5-(4-Chlorophenoxy)-3-methyl-1-phenyl-1H-pyrazol-4-yl]-1-[4-(prop-2-yn-1-yloxy)phenyl]prop-2-en-1-one (Ic) Special details

**Table:**

Geometry. All esds (except the esd in the dihedral angle between two l.s. planes) are estimated using the full covariance matrix. The cell esds are taken into account individually in the estimation of esds in distances, angles and torsion angles; correlations between esds in cell parameters are only used when they are defined by crystal symmetry. An approximate (isotropic) treatment of cell esds is used for estimating esds involving l.s. planes.

### 3-[5-(4-Chlorophenoxy)-3-methyl-1-phenyl-1H-pyrazol-4-yl]-1-[4-(prop-2-yn-1-yloxy)phenyl]prop-2-en-1-one (Ic) Fractional atomic coordinates and isotropic or equivalent isotropic displacement parameters (Å^2^)

**Table:**

	*x*	*y*	*z*	*U*_iso_*/*U*_eq_	
C1	0.5453 (2)	0.2711 (2)	0.53901 (14)	0.0354 (4)	
O1	0.49212 (18)	0.19336 (17)	0.46533 (11)	0.0535 (4)	
C2	0.5001 (2)	0.2494 (2)	0.63858 (14)	0.0375 (4)	
H2	0.5266	0.3202	0.6899	0.045*	
C3	0.4233 (2)	0.1329 (2)	0.65779 (14)	0.0360 (4)	
H3	0.3999	0.0635	0.6051	0.043*	
C11	0.65551 (19)	0.39198 (18)	0.52879 (13)	0.0321 (4)	
C12	0.6706 (2)	0.4355 (2)	0.43611 (13)	0.0366 (4)	
H12	0.6103	0.3890	0.3821	0.044*	
C13	0.7725 (2)	0.54596 (19)	0.42185 (14)	0.0373 (4)	
H13	0.7803	0.5739	0.3591	0.045*	
C14	0.8635 (2)	0.61513 (19)	0.50232 (14)	0.0351 (4)	
C15	0.8509 (2)	0.5724 (2)	0.59521 (14)	0.0430 (5)	
H15	0.9124	0.6180	0.6490	0.052*	
C16	0.7477 (2)	0.4627 (2)	0.60857 (14)	0.0408 (5)	
H16	0.7394	0.4355	0.6715	0.049*	
O14	0.96866 (16)	0.72487 (15)	0.49739 (10)	0.0466 (4)	
C17	0.9800 (2)	0.7763 (2)	0.40407 (16)	0.0456 (5)	
H17A	0.8869	0.8110	0.3832	0.055*	
H17B	0.9987	0.7023	0.3548	0.055*	
C18	1.1030 (2)	0.8878 (2)	0.41364 (16)	0.0445 (5)	
C19	1.2031 (3)	0.9760 (2)	0.41795 (18)	0.0541 (6)	
H19	1.2825	1.0460	0.4214	0.065*	
N31	0.32759 (17)	0.10603 (16)	0.91019 (11)	0.0359 (4)	
N32	0.23589 (18)	−0.00607 (17)	0.86251 (12)	0.0387 (4)	
C33	0.2648 (2)	−0.00897 (19)	0.76832 (14)	0.0350 (4)	
C34	0.37180 (19)	0.10233 (19)	0.75252 (13)	0.0324 (4)	
C35	0.4074 (2)	0.17216 (19)	0.84574 (13)	0.0333 (4)	
C311	0.3251 (2)	0.14030 (19)	1.01431 (14)	0.0376 (4)	
C312	0.4578 (3)	0.1772 (2)	1.07284 (16)	0.0498 (5)	
H312	0.5492	0.1809	1.0449	0.060*	
C313	0.4527 (3)	0.2086 (3)	1.17386 (18)	0.0639 (7)	
H313	0.5415	0.2351	1.2136	0.077*	
C314	0.3191 (4)	0.2013 (3)	1.21603 (18)	0.0660 (7)	
H314	0.3172	0.2223	1.2840	0.079*	
C315	0.1875 (3)	0.1625 (3)	1.15721 (19)	0.0633 (7)	
H315	0.0966	0.1564	1.1858	0.076*	
C316	0.1892 (3)	0.1326 (2)	1.05553 (16)	0.0478 (5)	
H316	0.1001	0.1077	1.0158	0.057*	
C331	0.1866 (3)	−0.1197 (2)	0.69277 (16)	0.0485 (5)	
H33A	0.1505	−0.1984	0.7248	0.073*	
H33B	0.2555	−0.1473	0.6458	0.073*	
H33C	0.1037	−0.0845	0.6591	0.073*	
O351	0.48920 (15)	0.29585 (13)	0.87828 (10)	0.0397 (3)	
C351	0.6443 (2)	0.31066 (19)	0.87288 (13)	0.0335 (4)	
C352	0.7128 (2)	0.4453 (2)	0.89176 (16)	0.0431 (5)	
H352	0.6565	0.5182	0.9075	0.052*	
C353	0.8669 (2)	0.4705 (2)	0.88698 (16)	0.0460 (5)	
H353	0.9148	0.5607	0.8994	0.055*	
C354	0.9481 (2)	0.3613 (2)	0.86373 (14)	0.0415 (5)	
Cl54	1.14061 (6)	0.39375 (8)	0.85575 (5)	0.0630 (2)	
C355	0.8803 (2)	0.2275 (2)	0.84574 (16)	0.0458 (5)	
H355	0.9369	0.1547	0.8305	0.055*	
C356	0.7260 (2)	0.2015 (2)	0.85043 (16)	0.0422 (5)	
H356	0.6785	0.1112	0.8385	0.051*	

### 3-[5-(4-Chlorophenoxy)-3-methyl-1-phenyl-1H-pyrazol-4-yl]-1-[4-(prop-2-yn-1-yloxy)phenyl]prop-2-en-1-one (Ic) Atomic displacement parameters (Å^2^)

**Table:**

	*U*^11^	*U*^22^	*U*^33^	*U*^12^	*U*^13^	*U*^23^
C1	0.0323 (9)	0.0385 (10)	0.0340 (9)	−0.0033 (7)	0.0052 (7)	0.0035 (8)
O1	0.0583 (9)	0.0560 (9)	0.0383 (8)	−0.0266 (7)	0.0049 (7)	0.0008 (7)
C2	0.0375 (10)	0.0407 (10)	0.0334 (9)	−0.0029 (8)	0.0059 (7)	0.0046 (8)
C3	0.0340 (9)	0.0396 (10)	0.0338 (9)	−0.0022 (8)	0.0067 (7)	0.0042 (8)
C11	0.0306 (9)	0.0326 (9)	0.0323 (9)	−0.0010 (7)	0.0053 (7)	0.0020 (7)
C12	0.0371 (10)	0.0393 (10)	0.0307 (9)	−0.0050 (8)	0.0015 (7)	0.0014 (7)
C13	0.0399 (10)	0.0376 (10)	0.0339 (9)	−0.0031 (8)	0.0041 (8)	0.0074 (8)
C14	0.0329 (9)	0.0306 (9)	0.0401 (10)	−0.0043 (7)	0.0071 (7)	0.0002 (7)
C15	0.0430 (11)	0.0469 (11)	0.0332 (10)	−0.0121 (9)	0.0010 (8)	−0.0040 (8)
C16	0.0433 (11)	0.0464 (11)	0.0300 (9)	−0.0092 (9)	0.0050 (8)	0.0045 (8)
O14	0.0492 (8)	0.0414 (8)	0.0439 (8)	−0.0179 (6)	0.0045 (6)	0.0028 (6)
C17	0.0423 (11)	0.0432 (11)	0.0492 (12)	−0.0097 (9)	0.0002 (9)	0.0125 (9)
C18	0.0425 (11)	0.0422 (11)	0.0486 (12)	−0.0027 (9)	0.0061 (9)	0.0095 (9)
C19	0.0508 (13)	0.0523 (13)	0.0564 (13)	−0.0144 (10)	0.0057 (10)	0.0119 (10)
N31	0.0378 (8)	0.0370 (8)	0.0320 (8)	−0.0017 (7)	0.0059 (6)	0.0035 (6)
N32	0.0384 (8)	0.0393 (9)	0.0370 (8)	−0.0047 (7)	0.0066 (7)	0.0040 (7)
C33	0.0336 (9)	0.0352 (9)	0.0357 (9)	−0.0014 (7)	0.0045 (7)	0.0059 (7)
C34	0.0305 (8)	0.0340 (9)	0.0330 (9)	0.0010 (7)	0.0051 (7)	0.0062 (7)
C35	0.0306 (9)	0.0330 (9)	0.0359 (9)	0.0003 (7)	0.0046 (7)	0.0033 (7)
C311	0.0488 (11)	0.0342 (9)	0.0317 (9)	0.0091 (8)	0.0076 (8)	0.0055 (7)
C312	0.0551 (13)	0.0560 (13)	0.0395 (11)	0.0168 (10)	0.0017 (9)	0.0015 (9)
C313	0.0885 (19)	0.0621 (15)	0.0411 (12)	0.0254 (14)	−0.0089 (12)	−0.0017 (11)
C314	0.111 (2)	0.0559 (15)	0.0355 (12)	0.0270 (15)	0.0143 (14)	0.0046 (10)
C315	0.0897 (19)	0.0548 (14)	0.0540 (14)	0.0181 (13)	0.0390 (14)	0.0136 (11)
C316	0.0553 (13)	0.0459 (12)	0.0455 (12)	0.0062 (10)	0.0177 (10)	0.0104 (9)
C331	0.0481 (12)	0.0482 (12)	0.0439 (11)	−0.0120 (9)	0.0045 (9)	−0.0027 (9)
O351	0.0352 (7)	0.0334 (7)	0.0484 (8)	−0.0014 (5)	0.0065 (6)	−0.0024 (6)
C351	0.0350 (9)	0.0334 (9)	0.0314 (9)	0.0010 (7)	0.0026 (7)	0.0027 (7)
C352	0.0432 (11)	0.0320 (10)	0.0518 (12)	0.0024 (8)	0.0019 (9)	−0.0028 (8)
C353	0.0460 (11)	0.0387 (11)	0.0490 (12)	−0.0073 (9)	−0.0020 (9)	0.0009 (9)
C354	0.0363 (10)	0.0530 (12)	0.0333 (10)	0.0000 (9)	0.0001 (8)	0.0024 (8)
Cl54	0.0371 (3)	0.0877 (5)	0.0605 (4)	−0.0021 (3)	0.0037 (2)	0.0001 (3)
C355	0.0422 (11)	0.0451 (11)	0.0500 (12)	0.0100 (9)	0.0022 (9)	0.0000 (9)
C356	0.0428 (11)	0.0324 (10)	0.0494 (11)	0.0010 (8)	0.0022 (9)	0.0002 (8)

### 3-[5-(4-Chlorophenoxy)-3-methyl-1-phenyl-1H-pyrazol-4-yl]-1-[4-(prop-2-yn-1-yloxy)phenyl]prop-2-en-1-one (Ic) Geometric parameters (Å, º)

**Table:**

C1—O1	1.229 (2)	C33—C331	1.493 (3)
C1—C2	1.474 (3)	C34—C35	1.378 (3)
C1—C11	1.483 (2)	C35—O351	1.360 (2)
C2—C3	1.327 (3)	C311—C316	1.380 (3)
C2—H2	0.9300	C311—C312	1.380 (3)
C3—C34	1.452 (2)	C312—C313	1.385 (3)
C3—H3	0.9300	C312—H312	0.9300
C11—C12	1.385 (3)	C313—C314	1.367 (4)
C11—C16	1.394 (3)	C313—H313	0.9300
C12—C13	1.378 (3)	C314—C315	1.377 (4)
C12—H12	0.9300	C314—H314	0.9300
C13—C14	1.390 (3)	C315—C316	1.389 (3)
C13—H13	0.9300	C315—H315	0.9300
C14—O14	1.362 (2)	C316—H316	0.9300
C14—C15	1.382 (3)	C331—H33A	0.9600
C15—C16	1.376 (3)	C331—H33B	0.9600
C15—H15	0.9300	C331—H33C	0.9600
C16—H16	0.9300	O351—C351	1.396 (2)
O14—C17	1.421 (2)	C351—C356	1.375 (3)
C17—C18	1.456 (3)	C351—C352	1.380 (3)
C17—H17A	0.9700	C352—C353	1.391 (3)
C17—H17B	0.9700	C352—H352	0.9300
C18—C19	1.172 (3)	C353—C354	1.374 (3)
C19—H19	0.9300	C353—H353	0.9300
N31—C35	1.347 (2)	C354—C355	1.370 (3)
N31—N32	1.373 (2)	C354—Cl54	1.741 (2)
N31—C311	1.427 (2)	C355—C356	1.393 (3)
N32—C33	1.327 (2)	C355—H355	0.9300
C33—C34	1.418 (2)	C356—H356	0.9300
			
O1—C1—C2	121.66 (17)	N31—C35—O351	118.65 (16)
O1—C1—C11	120.01 (16)	N31—C35—C34	108.52 (15)
C2—C1—C11	118.32 (16)	O351—C35—C34	132.30 (16)
C3—C2—C1	122.56 (18)	C316—C311—C312	120.78 (19)
C3—C2—H2	118.7	C316—C311—N31	119.17 (19)
C1—C2—H2	118.7	C312—C311—N31	120.03 (18)
C2—C3—C34	126.73 (18)	C311—C312—C313	119.0 (2)
C2—C3—H3	116.6	C311—C312—H312	120.5
C34—C3—H3	116.6	C313—C312—H312	120.5
C12—C11—C16	118.11 (16)	C314—C313—C312	121.0 (3)
C12—C11—C1	119.13 (16)	C314—C313—H313	119.5
C16—C11—C1	122.75 (16)	C312—C313—H313	119.5
C13—C12—C11	121.74 (17)	C313—C314—C315	119.5 (2)
C13—C12—H12	119.1	C313—C314—H314	120.2
C11—C12—H12	119.1	C315—C314—H314	120.2
C12—C13—C14	119.29 (17)	C314—C315—C316	120.6 (2)
C12—C13—H13	120.4	C314—C315—H315	119.7
C14—C13—H13	120.4	C316—C315—H315	119.7
O14—C14—C15	115.58 (16)	C311—C316—C315	119.0 (2)
O14—C14—C13	124.66 (17)	C311—C316—H316	120.5
C15—C14—C13	119.77 (16)	C315—C316—H316	120.5
C16—C15—C14	120.34 (17)	C33—C331—H33A	109.5
C16—C15—H15	119.8	C33—C331—H33B	109.5
C14—C15—H15	119.8	H33A—C331—H33B	109.5
C15—C16—C11	120.75 (17)	C33—C331—H33C	109.5
C15—C16—H16	119.6	H33A—C331—H33C	109.5
C11—C16—H16	119.6	H33B—C331—H33C	109.5
C14—O14—C17	117.27 (15)	C35—O351—C351	119.84 (14)
O14—C17—C18	108.51 (17)	C356—C351—C352	121.22 (18)
O14—C17—H17A	110.0	C356—C351—O351	123.80 (16)
C18—C17—H17A	110.0	C352—C351—O351	114.98 (16)
O14—C17—H17B	110.0	C351—C352—C353	119.14 (19)
C18—C17—H17B	110.0	C351—C352—H352	120.4
H17A—C17—H17B	108.4	C353—C352—H352	120.4
C19—C18—C17	177.6 (2)	C354—C353—C352	119.52 (19)
C18—C19—H19	180.0	C354—C353—H353	120.2
C35—N31—N32	110.96 (15)	C352—C353—H353	120.2
C35—N31—C311	129.06 (16)	C355—C354—C353	121.34 (19)
N32—N31—C311	119.96 (15)	C355—C354—Cl54	119.29 (17)
C33—N32—N31	104.91 (14)	C353—C354—Cl54	119.37 (16)
N32—C33—C34	112.02 (16)	C354—C355—C356	119.44 (19)
N32—C33—C331	120.58 (17)	C354—C355—H355	120.3
C34—C33—C331	127.39 (17)	C356—C355—H355	120.3
C35—C34—C33	103.56 (15)	C351—C356—C355	119.34 (18)
C35—C34—C3	130.35 (17)	C351—C356—H356	120.3
C33—C34—C3	126.06 (17)	C355—C356—H356	120.3
			
O1—C1—C2—C3	13.1 (3)	N32—N31—C35—C34	−1.2 (2)
C11—C1—C2—C3	−168.12 (19)	C311—N31—C35—C34	−179.14 (18)
C1—C2—C3—C34	−179.02 (18)	C33—C34—C35—N31	0.2 (2)
O1—C1—C11—C12	15.4 (3)	C3—C34—C35—N31	178.15 (19)
C2—C1—C11—C12	−163.41 (18)	C33—C34—C35—O351	−171.11 (19)
O1—C1—C11—C16	−163.3 (2)	C3—C34—C35—O351	6.8 (3)
C2—C1—C11—C16	17.9 (3)	C35—N31—C311—C316	136.4 (2)
C16—C11—C12—C13	−0.4 (3)	N32—N31—C311—C316	−41.4 (3)
C1—C11—C12—C13	−179.10 (18)	C35—N31—C311—C312	−45.3 (3)
C11—C12—C13—C14	0.4 (3)	N32—N31—C311—C312	137.0 (2)
C12—C13—C14—O14	179.64 (18)	C316—C311—C312—C313	−1.0 (3)
C12—C13—C14—C15	0.1 (3)	N31—C311—C312—C313	−179.3 (2)
O14—C14—C15—C16	179.76 (19)	C311—C312—C313—C314	1.1 (4)
C13—C14—C15—C16	−0.6 (3)	C312—C313—C314—C315	−0.2 (4)
C14—C15—C16—C11	0.7 (3)	C313—C314—C315—C316	−0.8 (4)
C12—C11—C16—C15	−0.2 (3)	C312—C311—C316—C315	0.0 (3)
C1—C11—C16—C15	178.48 (19)	N31—C311—C316—C315	178.35 (19)
C15—C14—O14—C17	−176.97 (19)	C314—C315—C316—C311	0.9 (3)
C13—C14—O14—C17	3.5 (3)	N31—C35—O351—C351	120.05 (18)
C14—O14—C17—C18	−177.14 (18)	C34—C35—O351—C351	−69.3 (3)
C35—N31—N32—C33	1.7 (2)	C35—O351—C351—C356	−10.4 (3)
C311—N31—N32—C33	179.86 (16)	C35—O351—C351—C352	169.43 (17)
N31—N32—C33—C34	−1.6 (2)	C356—C351—C352—C353	0.6 (3)
N31—N32—C33—C331	179.10 (18)	O351—C351—C352—C353	−179.26 (18)
N32—C33—C34—C35	0.9 (2)	C351—C352—C353—C354	−0.1 (3)
C331—C33—C34—C35	−179.9 (2)	C352—C353—C354—C355	−0.4 (3)
N32—C33—C34—C3	−177.16 (18)	C352—C353—C354—Cl54	178.92 (16)
C331—C33—C34—C3	2.1 (3)	C353—C354—C355—C356	0.4 (3)
C2—C3—C34—C35	−11.4 (3)	Cl54—C354—C355—C356	−178.93 (16)
C2—C3—C34—C33	166.1 (2)	C352—C351—C356—C355	−0.6 (3)
N32—N31—C35—O351	171.48 (15)	O351—C351—C356—C355	179.23 (18)
C311—N31—C35—O351	−6.4 (3)	C354—C355—C356—C351	0.1 (3)

### 3-[5-(4-Chlorophenoxy)-3-methyl-1-phenyl-1H-pyrazol-4-yl]-1-[4-(prop-2-yn-1-yloxy)phenyl]prop-2-en-1-one (Ic) Hydrogen-bond geometry (Å, º)

**Table:**

*D*—H···*A*	*D*—H	H···*A*	*D*···*A*	*D*—H···*A*
C19—H19···O1^i^	0.93	2.25	3.161 (3)	165
C16—H16···*Cg*2	0.93	2.98	3.882 (2)	165
C356—H356···*Cg*1^ii^	0.93	2.88	3.685 (2)	146

Symmetry codes: (i) *x*+1, *y*+1, *z*; (ii) −*x*+1, −*y*, −*z*+2.

### 3-[3-Methyl-5-(naphthalen-2-yloxy)-1-phenyl-1H-pyrazol-4-yl]-1-[4-(prop-2-ynyloxy)phenyl]prop-2-en-1-one (Ie) Crystal data

**Table:**

C_32_H_24_N_2_O_3_	*Z* = 2
*M**_r_* = 484.53	*F*(000) = 508
Triclinic, *P*1	*D*_x_ = 1.307 Mg m^−^^3^
*a* = 8.8615 (6) Å	Mo *K*α radiation, λ = 0.71073 Å
*b* = 10.4973 (7) Å	Cell parameters from 4376 reflections
*c* = 13.6588 (10) Å	θ = 3.0–25.2°
α = 79.006 (3)°	µ = 0.08 mm^−^^1^
β = 89.412 (3)°	*T* = 297 K
γ = 80.971 (3)°	Block, brown
*V* = 1231.54 (15) Å^3^	0.20 × 0.16 × 0.16 mm

### 3-[3-Methyl-5-(naphthalen-2-yloxy)-1-phenyl-1H-pyrazol-4-yl]-1-[4-(prop-2-ynyloxy)phenyl]prop-2-en-1-one (Ie) Data collection

**Table:**

Bruker APEXII diffractometer	4373 independent reflections
Radiation source: fine focussealed tube	3474 reflections with *I* > 2σ(*I*)
Graphite monochromator	*R*_int_ = 0.055
φ and ω scans	θ_max_ = 25.2°, θ_min_ = 3.4°
Absorption correction: multi-scan (SADABS; Bruker, 2016)	*h* = −10→10
*T*_min_ = 0.898, *T*_max_ = 0.987	*k* = −12→12
30613 measured reflections	*l* = −16→16

### 3-[3-Methyl-5-(naphthalen-2-yloxy)-1-phenyl-1H-pyrazol-4-yl]-1-[4-(prop-2-ynyloxy)phenyl]prop-2-en-1-one (Ie) Refinement

**Table:**

Refinement on *F*^2^	Hydrogen site location: inferred from neighbouring sites
Least-squares matrix: full	H-atom parameters constrained
*R*[*F*^2^ > 2σ(*F*^2^)] = 0.070	*w* = 1/[σ^2^(*F*_o_^2^) + (0.0694*P*)^2^ + 1.3194*P*] where *P* = (*F*_o_^2^ + 2*F*_c_^2^)/3
*wR*(*F*^2^) = 0.187	(Δ/σ)_max_ < 0.001
*S* = 1.07	Δρ_max_ = 0.85 e Å^−^^3^
4373 reflections	Δρ_min_ = −0.29 e Å^−^^3^
336 parameters	Extinction correction: SHELXL, Fc^*^=kFc[1+0.001xFc^2^λ^3^/sin(2θ)]^-1/4^
0 restraints	Extinction coefficient: 0.114 (12)
Primary atom site location: dual	

### 3-[3-Methyl-5-(naphthalen-2-yloxy)-1-phenyl-1H-pyrazol-4-yl]-1-[4-(prop-2-ynyloxy)phenyl]prop-2-en-1-one (Ie) Special details

**Table:**

Geometry. All esds (except the esd in the dihedral angle between two l.s. planes) are estimated using the full covariance matrix. The cell esds are taken into account individually in the estimation of esds in distances, angles and torsion angles; correlations between esds in cell parameters are only used when they are defined by crystal symmetry. An approximate (isotropic) treatment of cell esds is used for estimating esds involving l.s. planes.

### 3-[3-Methyl-5-(naphthalen-2-yloxy)-1-phenyl-1H-pyrazol-4-yl]-1-[4-(prop-2-ynyloxy)phenyl]prop-2-en-1-one (Ie) Fractional atomic coordinates and isotropic or equivalent isotropic displacement parameters (Å^2^)

**Table:**

	*x*	*y*	*z*	*U*_iso_*/*U*_eq_	
C1	0.4597 (3)	0.2692 (3)	0.9522 (2)	0.0466 (6)	
O1	0.5161 (3)	0.1921 (2)	1.02722 (15)	0.0682 (7)	
C2	0.5008 (3)	0.2472 (3)	0.8514 (2)	0.0479 (7)	
H2	0.4710	0.3145	0.7970	0.057*	
C3	0.5789 (3)	0.1344 (3)	0.8354 (2)	0.0456 (6)	
H3	0.6058	0.0694	0.8916	0.055*	
C11	0.3503 (3)	0.3873 (3)	0.96264 (18)	0.0421 (6)	
C12	0.3407 (3)	0.4290 (3)	1.0532 (2)	0.0491 (7)	
H12	0.4032	0.3815	1.1063	0.059*	
C13	0.2408 (3)	0.5392 (3)	1.0669 (2)	0.0501 (7)	
H13	0.2376	0.5664	1.1279	0.060*	
C14	0.1454 (3)	0.6086 (3)	0.9883 (2)	0.0463 (6)	
C15	0.1510 (3)	0.5671 (3)	0.8978 (2)	0.0539 (7)	
H15	0.0854	0.6126	0.8457	0.065*	
C16	0.2528 (3)	0.4595 (3)	0.8848 (2)	0.0516 (7)	
H16	0.2572	0.4340	0.8232	0.062*	
O14	0.0419 (2)	0.7179 (2)	0.99303 (15)	0.0600 (6)	
C17	0.0309 (4)	0.7627 (3)	1.0852 (2)	0.0584 (8)	
H17A	0.1258	0.7910	1.0999	0.070*	
H17B	0.0129	0.6918	1.1387	0.070*	
C18	−0.0948 (4)	0.8721 (3)	1.0781 (2)	0.0584 (8)	
C19	−0.1963 (4)	0.9573 (4)	1.0761 (3)	0.0695 (9)	
H19	−0.2776	1.0256	1.0745	0.083*	
N31	0.6678 (2)	0.0973 (2)	0.58075 (16)	0.0441 (5)	
N32	0.7581 (3)	−0.0141 (2)	0.63236 (16)	0.0481 (6)	
C33	0.7313 (3)	−0.0118 (3)	0.72742 (19)	0.0439 (6)	
C34	0.6271 (3)	0.1007 (3)	0.74080 (19)	0.0415 (6)	
C35	0.5907 (3)	0.1663 (2)	0.64457 (19)	0.0423 (6)	
C311	0.6675 (3)	0.1256 (3)	0.47431 (19)	0.0435 (6)	
C312	0.5324 (3)	0.1747 (3)	0.4211 (2)	0.0591 (8)	
H312	0.4408	0.1889	0.4542	0.071*	
C313	0.5349 (4)	0.2025 (4)	0.3179 (2)	0.0681 (9)	
H313	0.4444	0.2372	0.2819	0.082*	
C314	0.6679 (4)	0.1798 (3)	0.2679 (2)	0.0631 (9)	
H314	0.6683	0.1990	0.1986	0.076*	
C315	0.8004 (4)	0.1285 (3)	0.3216 (2)	0.0640 (9)	
H315	0.8910	0.1114	0.2881	0.077*	
C316	0.8019 (3)	0.1014 (3)	0.4253 (2)	0.0535 (7)	
H316	0.8927	0.0674	0.4610	0.064*	
C331	0.8082 (4)	−0.1185 (3)	0.8078 (2)	0.0595 (8)	
H33A	0.8606	−0.1889	0.7786	0.089*	
H33B	0.7331	−0.1508	0.8529	0.089*	
H33C	0.8805	−0.0843	0.8435	0.089*	
O351	0.5097 (2)	0.28760 (18)	0.60811 (15)	0.0528 (5)	
C351	0.2781 (4)	0.4338 (3)	0.6115 (2)	0.0550 (7)	
H351	0.3356	0.5017	0.5971	0.066*	
C352	0.3475 (3)	0.3071 (3)	0.62206 (19)	0.0507 (7)	
C353	0.2669 (4)	0.2018 (3)	0.6420 (2)	0.0595 (8)	
H353	0.3175	0.1160	0.6483	0.071*	
C354	0.1103 (4)	0.2270 (3)	0.6523 (3)	0.0669 (9)	
H354	0.0553	0.1573	0.6654	0.080*	
C355	−0.1287 (4)	0.3815 (4)	0.6550 (3)	0.0695 (10)	
H355	−0.1864	0.3137	0.6686	0.083*	
C356	−0.1961 (4)	0.5079 (4)	0.6457 (3)	0.0737 (10)	
H356	−0.3013	0.5257	0.6533	0.088*	
C357	−0.1145 (5)	0.6132 (4)	0.6250 (3)	0.0791 (11)	
H357	−0.1648	0.6991	0.6188	0.095*	
C358	0.0361 (4)	0.5891 (4)	0.6142 (3)	0.0722 (10)	
H358	0.0898	0.6596	0.6005	0.087*	
C359	0.0346 (4)	0.3537 (3)	0.6434 (2)	0.0589 (8)	
C360	0.1159 (3)	0.4629 (3)	0.62260 (19)	0.0447 (6)	

### 3-[3-Methyl-5-(naphthalen-2-yloxy)-1-phenyl-1H-pyrazol-4-yl]-1-[4-(prop-2-ynyloxy)phenyl]prop-2-en-1-one (Ie) Atomic displacement parameters (Å^2^)

**Table:**

	*U*^11^	*U*^22^	*U*^33^	*U*^12^	*U*^13^	*U*^23^
C1	0.0466 (15)	0.0465 (15)	0.0434 (15)	0.0048 (12)	−0.0025 (12)	−0.0100 (12)
O1	0.0799 (15)	0.0664 (14)	0.0440 (12)	0.0306 (12)	−0.0081 (10)	−0.0086 (10)
C2	0.0506 (16)	0.0482 (15)	0.0404 (14)	0.0053 (12)	−0.0009 (12)	−0.0081 (12)
C3	0.0443 (14)	0.0458 (15)	0.0430 (14)	0.0025 (12)	−0.0013 (11)	−0.0073 (11)
C11	0.0435 (14)	0.0418 (14)	0.0369 (13)	0.0038 (11)	−0.0001 (11)	−0.0057 (11)
C12	0.0500 (16)	0.0516 (16)	0.0394 (14)	0.0096 (13)	−0.0072 (12)	−0.0066 (12)
C13	0.0572 (17)	0.0491 (16)	0.0403 (14)	0.0077 (13)	−0.0031 (12)	−0.0125 (12)
C14	0.0474 (15)	0.0408 (14)	0.0455 (15)	0.0065 (11)	0.0018 (12)	−0.0064 (11)
C15	0.0555 (17)	0.0577 (17)	0.0395 (15)	0.0138 (14)	−0.0085 (12)	−0.0047 (12)
C16	0.0578 (17)	0.0541 (17)	0.0371 (14)	0.0104 (13)	−0.0051 (12)	−0.0098 (12)
O14	0.0669 (13)	0.0537 (12)	0.0499 (12)	0.0219 (10)	−0.0050 (10)	−0.0115 (9)
C17	0.0588 (18)	0.0567 (18)	0.0560 (18)	0.0116 (14)	−0.0046 (14)	−0.0186 (14)
C18	0.0595 (18)	0.0551 (18)	0.0574 (18)	0.0067 (15)	0.0010 (14)	−0.0154 (14)
C19	0.069 (2)	0.069 (2)	0.065 (2)	0.0181 (18)	0.0005 (16)	−0.0209 (17)
N31	0.0445 (12)	0.0460 (12)	0.0375 (12)	0.0039 (10)	−0.0017 (9)	−0.0063 (9)
N32	0.0472 (13)	0.0491 (13)	0.0411 (12)	0.0104 (10)	−0.0011 (10)	−0.0058 (10)
C33	0.0415 (14)	0.0466 (15)	0.0399 (14)	0.0034 (11)	−0.0016 (11)	−0.0070 (11)
C34	0.0405 (13)	0.0416 (14)	0.0402 (14)	0.0016 (11)	−0.0006 (11)	−0.0093 (11)
C35	0.0400 (13)	0.0393 (13)	0.0442 (14)	0.0031 (11)	−0.0019 (11)	−0.0073 (11)
C311	0.0474 (15)	0.0425 (14)	0.0391 (14)	−0.0045 (11)	−0.0006 (11)	−0.0063 (11)
C312	0.0474 (16)	0.080 (2)	0.0448 (16)	−0.0008 (15)	−0.0030 (13)	−0.0073 (15)
C313	0.068 (2)	0.082 (2)	0.0475 (18)	−0.0024 (18)	−0.0128 (15)	−0.0033 (16)
C314	0.088 (2)	0.0583 (19)	0.0422 (16)	−0.0131 (17)	0.0016 (16)	−0.0058 (14)
C315	0.071 (2)	0.064 (2)	0.0561 (19)	−0.0086 (16)	0.0194 (16)	−0.0115 (15)
C316	0.0483 (16)	0.0547 (17)	0.0538 (17)	0.0009 (13)	0.0028 (13)	−0.0084 (13)
C331	0.0629 (19)	0.0568 (18)	0.0476 (16)	0.0169 (15)	0.0012 (14)	−0.0034 (13)
O351	0.0560 (12)	0.0400 (10)	0.0550 (12)	0.0056 (9)	−0.0027 (9)	−0.0010 (8)
C351	0.0598 (18)	0.0475 (16)	0.0546 (17)	0.0006 (13)	−0.0104 (14)	−0.0086 (13)
C352	0.0560 (17)	0.0508 (16)	0.0369 (14)	0.0199 (13)	−0.0108 (12)	−0.0100 (12)
C353	0.0554 (18)	0.0525 (17)	0.067 (2)	−0.0013 (14)	−0.0014 (15)	−0.0078 (14)
C354	0.064 (2)	0.0564 (19)	0.078 (2)	−0.0105 (16)	−0.0048 (17)	−0.0048 (16)
C355	0.0440 (17)	0.097 (3)	0.064 (2)	−0.0023 (17)	−0.0037 (14)	−0.0123 (18)
C356	0.0541 (19)	0.099 (3)	0.061 (2)	0.009 (2)	−0.0034 (16)	−0.0163 (19)
C357	0.077 (3)	0.087 (3)	0.065 (2)	0.025 (2)	−0.0158 (18)	−0.0249 (19)
C358	0.084 (3)	0.062 (2)	0.065 (2)	0.0198 (18)	−0.0179 (18)	−0.0212 (16)
C359	0.0627 (19)	0.067 (2)	0.0421 (16)	0.0060 (15)	−0.0094 (13)	−0.0100 (14)
C360	0.0479 (15)	0.0489 (15)	0.0351 (13)	0.0018 (12)	−0.0072 (11)	−0.0094 (11)

### 3-[3-Methyl-5-(naphthalen-2-yloxy)-1-phenyl-1H-pyrazol-4-yl]-1-[4-(prop-2-ynyloxy)phenyl]prop-2-en-1-one (Ie) Geometric parameters (Å, º)

**Table:**

C1—O1	1.233 (3)	C311—C312	1.382 (4)
C1—C2	1.472 (4)	C312—C313	1.384 (4)
C1—C11	1.478 (4)	C312—H312	0.9300
C2—C3	1.330 (4)	C313—C314	1.367 (5)
C2—H2	0.9300	C313—H313	0.9300
C3—C34	1.446 (4)	C314—C315	1.369 (5)
C3—H3	0.9300	C314—H314	0.9300
C11—C12	1.385 (4)	C315—C316	1.390 (4)
C11—C16	1.399 (4)	C315—H315	0.9300
C12—C13	1.384 (4)	C316—H316	0.9300
C12—H12	0.9300	C331—H33A	0.9600
C13—C14	1.387 (4)	C331—H33B	0.9600
C13—H13	0.9300	C331—H33C	0.9600
C14—O14	1.363 (3)	O351—C352	1.435 (3)
C14—C15	1.384 (4)	C351—C352	1.356 (4)
C15—C16	1.370 (4)	C351—C360	1.434 (4)
C15—H15	0.9300	C351—H351	0.9300
C16—H16	0.9300	C352—C353	1.391 (4)
O14—C17	1.423 (3)	C353—C354	1.382 (5)
C17—C18	1.459 (4)	C353—H353	0.9300
C17—H17A	0.9700	C354—C359	1.376 (5)
C17—H17B	0.9700	C354—H354	0.9300
C18—C19	1.161 (4)	C355—C356	1.350 (5)
C19—H19	0.9300	C355—C359	1.444 (4)
N31—C35	1.351 (3)	C355—H355	0.9300
N31—N32	1.382 (3)	C356—C357	1.397 (6)
N31—C311	1.427 (3)	C356—H356	0.9300
N32—C33	1.322 (3)	C357—C358	1.331 (5)
C33—C34	1.420 (4)	C357—H357	0.9300
C33—C331	1.494 (4)	C358—C360	1.385 (4)
C34—C35	1.378 (4)	C358—H358	0.9300
C35—O351	1.365 (3)	C359—C360	1.430 (4)
C311—C316	1.372 (4)		
			
O1—C1—C2	121.3 (2)	C311—C312—C313	119.2 (3)
O1—C1—C11	120.0 (2)	C311—C312—H312	120.4
C2—C1—C11	118.8 (2)	C313—C312—H312	120.4
C3—C2—C1	122.2 (3)	C314—C313—C312	121.2 (3)
C3—C2—H2	118.9	C314—C313—H313	119.4
C1—C2—H2	118.9	C312—C313—H313	119.4
C2—C3—C34	127.7 (3)	C313—C314—C315	119.0 (3)
C2—C3—H3	116.2	C313—C314—H314	120.5
C34—C3—H3	116.2	C315—C314—H314	120.5
C12—C11—C16	117.6 (2)	C314—C315—C316	121.2 (3)
C12—C11—C1	119.5 (2)	C314—C315—H315	119.4
C16—C11—C1	122.8 (2)	C316—C315—H315	119.4
C13—C12—C11	121.9 (2)	C311—C316—C315	119.1 (3)
C13—C12—H12	119.0	C311—C316—H316	120.4
C11—C12—H12	119.0	C315—C316—H316	120.4
C12—C13—C14	119.1 (3)	C33—C331—H33A	109.5
C12—C13—H13	120.5	C33—C331—H33B	109.5
C14—C13—H13	120.5	H33A—C331—H33B	109.5
O14—C14—C15	115.7 (2)	C33—C331—H33C	109.5
O14—C14—C13	124.4 (2)	H33A—C331—H33C	109.5
C15—C14—C13	119.9 (2)	H33B—C331—H33C	109.5
C16—C15—C14	120.2 (2)	C35—O351—C352	118.0 (2)
C16—C15—H15	119.9	C352—C351—C360	119.9 (3)
C14—C15—H15	119.9	C352—C351—H351	120.0
C15—C16—C11	121.2 (3)	C360—C351—H351	120.0
C15—C16—H16	119.4	C351—C352—C353	122.5 (3)
C11—C16—H16	119.4	C351—C352—O351	116.0 (3)
C14—O14—C17	117.5 (2)	C353—C352—O351	121.5 (2)
O14—C17—C18	109.2 (2)	C354—C353—C352	118.9 (3)
O14—C17—H17A	109.8	C354—C353—H353	120.6
C18—C17—H17A	109.8	C352—C353—H353	120.6
O14—C17—H17B	109.8	C359—C354—C353	121.0 (3)
C18—C17—H17B	109.8	C359—C354—H354	119.5
H17A—C17—H17B	108.3	C353—C354—H354	119.5
C19—C18—C17	177.5 (4)	C356—C355—C359	118.8 (4)
C18—C19—H19	180.0	C356—C355—H355	120.6
C35—N31—N32	110.6 (2)	C359—C355—H355	120.6
C35—N31—C311	129.4 (2)	C355—C356—C357	122.7 (3)
N32—N31—C311	119.9 (2)	C355—C356—H356	118.6
C33—N32—N31	104.8 (2)	C357—C356—H356	118.6
N32—C33—C34	112.5 (2)	C358—C357—C356	119.3 (4)
N32—C33—C331	120.9 (2)	C358—C357—H357	120.4
C34—C33—C331	126.6 (2)	C356—C357—H357	120.4
C35—C34—C33	103.3 (2)	C357—C358—C360	122.4 (4)
C35—C34—C3	130.6 (2)	C357—C358—H358	118.8
C33—C34—C3	126.0 (2)	C360—C358—H358	118.8
N31—C35—O351	119.2 (2)	C354—C359—C360	120.8 (3)
N31—C35—C34	108.7 (2)	C354—C359—C355	121.6 (3)
O351—C35—C34	131.6 (2)	C360—C359—C355	117.6 (3)
C316—C311—C312	120.3 (3)	C358—C360—C359	119.3 (3)
C316—C311—N31	119.5 (2)	C358—C360—C351	123.7 (3)
C312—C311—N31	120.2 (2)	C359—C360—C351	117.0 (3)
			
O1—C1—C2—C3	11.5 (5)	C3—C34—C35—O351	6.6 (5)
C11—C1—C2—C3	−169.4 (3)	C35—N31—C311—C316	140.2 (3)
C1—C2—C3—C34	−179.5 (3)	N32—N31—C311—C316	−39.0 (4)
O1—C1—C11—C12	16.3 (4)	C35—N31—C311—C312	−40.9 (4)
C2—C1—C11—C12	−162.8 (3)	N32—N31—C311—C312	139.9 (3)
O1—C1—C11—C16	−162.8 (3)	C316—C311—C312—C313	−1.7 (5)
C2—C1—C11—C16	18.0 (4)	N31—C311—C312—C313	179.4 (3)
C16—C11—C12—C13	−1.1 (4)	C311—C312—C313—C314	1.3 (5)
C1—C11—C12—C13	179.7 (3)	C312—C313—C314—C315	0.1 (5)
C11—C12—C13—C14	1.3 (5)	C313—C314—C315—C316	−1.1 (5)
C12—C13—C14—O14	179.6 (3)	C312—C311—C316—C315	0.7 (4)
C12—C13—C14—C15	0.1 (5)	N31—C311—C316—C315	179.6 (3)
O14—C14—C15—C16	179.0 (3)	C314—C315—C316—C311	0.7 (5)
C13—C14—C15—C16	−1.5 (5)	N31—C35—O351—C352	118.5 (3)
C14—C15—C16—C11	1.6 (5)	C34—C35—O351—C352	−70.6 (4)
C12—C11—C16—C15	−0.3 (5)	C360—C351—C352—C353	1.1 (4)
C1—C11—C16—C15	178.8 (3)	C360—C351—C352—O351	179.0 (2)
C15—C14—O14—C17	179.1 (3)	C35—O351—C352—C351	161.8 (2)
C13—C14—O14—C17	−0.4 (4)	C35—O351—C352—C353	−20.2 (4)
C14—O14—C17—C18	−174.7 (3)	C351—C352—C353—C354	−0.5 (5)
C35—N31—N32—C33	1.2 (3)	O351—C352—C353—C354	−178.4 (3)
C311—N31—N32—C33	−179.5 (2)	C352—C353—C354—C359	−0.2 (5)
N31—N32—C33—C34	−1.3 (3)	C359—C355—C356—C357	0.2 (5)
N31—N32—C33—C331	179.5 (3)	C355—C356—C357—C358	−0.2 (6)
N32—C33—C34—C35	0.8 (3)	C356—C357—C358—C360	0.1 (5)
C331—C33—C34—C35	−180.0 (3)	C353—C354—C359—C360	0.5 (5)
N32—C33—C34—C3	−177.6 (3)	C353—C354—C359—C355	−179.4 (3)
C331—C33—C34—C3	1.6 (5)	C356—C355—C359—C354	179.8 (3)
C2—C3—C34—C35	−9.1 (5)	C356—C355—C359—C360	0.0 (5)
C2—C3—C34—C33	168.9 (3)	C357—C358—C360—C359	0.1 (5)
N32—N31—C35—O351	172.1 (2)	C357—C358—C360—C351	−179.9 (3)
C311—N31—C35—O351	−7.1 (4)	C354—C359—C360—C358	−180.0 (3)
N32—N31—C35—C34	−0.7 (3)	C355—C359—C360—C358	−0.2 (4)
C311—N31—C35—C34	−180.0 (3)	C354—C359—C360—C351	0.0 (4)
C33—C34—C35—N31	−0.1 (3)	C355—C359—C360—C351	179.9 (3)
C3—C34—C35—N31	178.3 (3)	C352—C351—C360—C358	179.2 (3)
C33—C34—C35—O351	−171.7 (3)	C352—C351—C360—C359	−0.8 (4)

### 3-[3-Methyl-5-(naphthalen-2-yloxy)-1-phenyl-1H-pyrazol-4-yl]-1-[4-(prop-2-ynyloxy)phenyl]prop-2-en-1-one (Ie) Hydrogen-bond geometry (Å, º)

**Table:**

*D*—H···*A*	*D*—H	H···*A*	*D*···*A*	*D*—H···*A*
C19—H19···O1^i^	0.93	2.32	3.233 (5)	165
C353—H353···*Cg*1^ii^	0.93	2.86	3.708 (3)	152

Symmetry codes: (i) *x*−1, *y*+1, *z*; (ii) −*x*+1, −*y*, −*z*+1.

### 1-(4-Azidophenyl)-3-[3-methyl-5-(2-methylphenoxy)-1-phenyl-1H-pyrazol-4-yl]prop-2-en-1-one (IIa) Crystal data

**Table:**

C_26_H_21_N_5_O_2_	*Z* = 2
*M**_r_* = 435.48	*F*(000) = 456
Triclinic, *P*1	*D*_x_ = 1.255 Mg m^−^^3^
*a* = 9.8432 (6) Å	Mo *K*α radiation, λ = 0.71073 Å
*b* = 11.7441 (7) Å	Cell parameters from 4051 reflections
*c* = 12.3005 (7) Å	θ = 3.2–25.1°
α = 114.120 (2)°	µ = 0.08 mm^−^^1^
β = 111.139 (2)°	*T* = 297 K
γ = 96.537 (2)°	Block, brown
*V* = 1152.06 (12) Å^3^	0.20 × 0.20 × 0.18 mm

### 1-(4-Azidophenyl)-3-[3-methyl-5-(2-methylphenoxy)-1-phenyl-1H-pyrazol-4-yl]prop-2-en-1-one (IIa) Data collection

**Table:**

Bruker APEXII diffractometer	4050 independent reflections
Radiation source: fine focussealed tube	2957 reflections with *I* > 2σ(*I*)
Graphite monochromator	*R*_int_ = 0.048
φ and ω scans	θ_max_ = 25.1°, θ_min_ = 3.5°
Absorption correction: multi-scan (SADABS; Bruker, 2016)	*h* = −11→11
*T*_min_ = 0.868, *T*_max_ = 0.985	*k* = −13→13
17379 measured reflections	*l* = −14→14

### 1-(4-Azidophenyl)-3-[3-methyl-5-(2-methylphenoxy)-1-phenyl-1H-pyrazol-4-yl]prop-2-en-1-one (IIa) Refinement

**Table:**

Refinement on *F*^2^	Hydrogen site location: inferred from neighbouring sites
Least-squares matrix: full	H-atom parameters constrained
*R*[*F*^2^ > 2σ(*F*^2^)] = 0.050	*w* = 1/[σ^2^(*F*_o_^2^) + (0.0547*P*)^2^ + 0.4937*P*] where *P* = (*F*_o_^2^ + 2*F*_c_^2^)/3
*wR*(*F*^2^) = 0.148	(Δ/σ)_max_ < 0.001
*S* = 1.10	Δρ_max_ = 0.19 e Å^−^^3^
4050 reflections	Δρ_min_ = −0.21 e Å^−^^3^
301 parameters	Extinction correction: SHELXL, Fc^*^=kFc[1+0.001xFc^2^λ^3^/sin(2θ)]^-1/4^
0 restraints	Extinction coefficient: 0.179 (14)
Primary atom site location: dual	

### 1-(4-Azidophenyl)-3-[3-methyl-5-(2-methylphenoxy)-1-phenyl-1H-pyrazol-4-yl]prop-2-en-1-one (IIa) Special details

**Table:**

Geometry. All esds (except the esd in the dihedral angle between two l.s. planes) are estimated using the full covariance matrix. The cell esds are taken into account individually in the estimation of esds in distances, angles and torsion angles; correlations between esds in cell parameters are only used when they are defined by crystal symmetry. An approximate (isotropic) treatment of cell esds is used for estimating esds involving l.s. planes.

### 1-(4-Azidophenyl)-3-[3-methyl-5-(2-methylphenoxy)-1-phenyl-1H-pyrazol-4-yl]prop-2-en-1-one (IIa) Fractional atomic coordinates and isotropic or equivalent isotropic displacement parameters (Å^2^)

**Table:**

	*x*	*y*	*z*	*U*_iso_*/*U*_eq_	
C1	0.6025 (2)	0.4013 (2)	0.8884 (2)	0.0473 (5)	
O1	0.66955 (18)	0.33036 (17)	0.9214 (2)	0.0733 (5)	
C2	0.4353 (2)	0.3679 (2)	0.8333 (2)	0.0465 (5)	
H2	0.3862	0.4229	0.8090	0.056*	
C3	0.3533 (2)	0.2591 (2)	0.8180 (2)	0.0459 (5)	
H3	0.4098	0.2111	0.8489	0.055*	
C11	0.6922 (2)	0.52181 (19)	0.90264 (19)	0.0415 (5)	
C12	0.8501 (2)	0.5646 (2)	0.9800 (2)	0.0462 (5)	
H12	0.8940	0.5193	1.0229	0.055*	
C13	0.9434 (2)	0.6725 (2)	0.9947 (2)	0.0471 (5)	
H13	1.0487	0.6991	1.0463	0.057*	
C14	0.8781 (2)	0.7402 (2)	0.9317 (2)	0.0480 (5)	
C15	0.7212 (2)	0.7008 (2)	0.8556 (2)	0.0557 (6)	
H15	0.6776	0.7473	0.8142	0.067*	
C16	0.6292 (2)	0.5923 (2)	0.8413 (2)	0.0519 (5)	
H16	0.5239	0.5662	0.7900	0.062*	
N14	0.9637 (2)	0.85203 (19)	0.9401 (2)	0.0656 (6)	
N15	1.1040 (3)	0.8907 (2)	1.0131 (2)	0.0693 (6)	
N16	1.2312 (3)	0.9373 (3)	1.0751 (3)	0.1029 (10)	
N31	−0.06215 (19)	0.16164 (16)	0.66089 (17)	0.0471 (4)	
N32	−0.0398 (2)	0.05926 (17)	0.68563 (18)	0.0511 (5)	
C33	0.1109 (2)	0.0864 (2)	0.7460 (2)	0.0469 (5)	
C34	0.1897 (2)	0.20522 (19)	0.7604 (2)	0.0433 (5)	
C35	0.0727 (2)	0.24912 (19)	0.7052 (2)	0.0442 (5)	
C311	−0.2141 (2)	0.1559 (2)	0.5825 (2)	0.0471 (5)	
C312	−0.2664 (3)	0.2627 (2)	0.6210 (3)	0.0611 (6)	
H312	−0.2034	0.3406	0.6988	0.073*	
C313	−0.4142 (3)	0.2524 (3)	0.5418 (3)	0.0711 (7)	
H313	−0.4506	0.3239	0.5667	0.085*	
C314	−0.5075 (3)	0.1371 (3)	0.4269 (3)	0.0735 (7)	
H314	−0.6063	0.1312	0.3741	0.088*	
C315	−0.4550 (3)	0.0308 (3)	0.3900 (3)	0.0719 (7)	
H315	−0.5184	−0.0472	0.3124	0.086*	
C316	−0.3082 (3)	0.0397 (2)	0.4679 (2)	0.0578 (6)	
H316	−0.2728	−0.0324	0.4433	0.069*	
C331	0.1796 (3)	−0.0017 (2)	0.7929 (3)	0.0645 (6)	
H33A	0.1019	−0.0622	0.7896	0.097*	
H33B	0.2587	0.0498	0.8829	0.097*	
H33C	0.2225	−0.0495	0.7365	0.097*	
O351	0.07382 (17)	0.36198 (13)	0.69897 (15)	0.0522 (4)	
C351	0.1292 (2)	0.38472 (19)	0.6168 (2)	0.0434 (5)	
C352	0.1738 (2)	0.5152 (2)	0.6479 (2)	0.0522 (5)	
C353	0.2282 (3)	0.5403 (3)	0.5678 (3)	0.0690 (7)	
H353	0.2599	0.6264	0.5855	0.083*	
C354	0.2367 (3)	0.4418 (3)	0.4633 (3)	0.0780 (8)	
H354	0.2741	0.4618	0.4116	0.094*	
C355	0.1900 (3)	0.3135 (3)	0.4344 (3)	0.0699 (7)	
H355	0.1952	0.2469	0.3630	0.084*	
C356	0.1353 (3)	0.2839 (2)	0.5119 (2)	0.0547 (6)	
H356	0.1032	0.1976	0.4934	0.066*	
C357	0.1657 (4)	0.6222 (2)	0.7625 (3)	0.0777 (8)	
H35A	0.1876	0.7031	0.7608	0.116*	
H35B	0.2393	0.6314	0.8446	0.116*	
H35C	0.0649	0.6010	0.7559	0.116*	

### 1-(4-Azidophenyl)-3-[3-methyl-5-(2-methylphenoxy)-1-phenyl-1H-pyrazol-4-yl]prop-2-en-1-one (IIa) Atomic displacement parameters (Å^2^)

**Table:**

	*U*^11^	*U*^22^	*U*^33^	*U*^12^	*U*^13^	*U*^23^
C1	0.0414 (11)	0.0494 (12)	0.0507 (12)	0.0123 (9)	0.0182 (9)	0.0266 (10)
O1	0.0471 (9)	0.0702 (11)	0.1105 (14)	0.0170 (8)	0.0231 (9)	0.0615 (11)
C2	0.0378 (11)	0.0456 (11)	0.0520 (12)	0.0110 (9)	0.0178 (9)	0.0227 (9)
C3	0.0441 (11)	0.0436 (11)	0.0474 (11)	0.0122 (9)	0.0214 (9)	0.0194 (9)
C11	0.0362 (10)	0.0439 (11)	0.0414 (10)	0.0110 (8)	0.0166 (8)	0.0192 (9)
C12	0.0382 (11)	0.0490 (12)	0.0531 (12)	0.0147 (9)	0.0193 (9)	0.0270 (10)
C13	0.0364 (10)	0.0470 (11)	0.0531 (12)	0.0111 (9)	0.0171 (9)	0.0231 (10)
C14	0.0467 (12)	0.0430 (11)	0.0500 (12)	0.0074 (9)	0.0213 (9)	0.0207 (9)
C15	0.0478 (12)	0.0569 (13)	0.0614 (13)	0.0119 (10)	0.0157 (10)	0.0366 (11)
C16	0.0375 (11)	0.0573 (13)	0.0544 (12)	0.0091 (9)	0.0127 (9)	0.0298 (11)
N14	0.0517 (12)	0.0558 (12)	0.0775 (14)	0.0002 (9)	0.0136 (10)	0.0397 (11)
N15	0.0585 (14)	0.0588 (13)	0.0799 (14)	0.0032 (10)	0.0187 (12)	0.0388 (11)
N16	0.0565 (15)	0.0956 (19)	0.130 (2)	−0.0054 (13)	0.0099 (15)	0.0668 (18)
N31	0.0417 (9)	0.0424 (9)	0.0551 (10)	0.0076 (7)	0.0203 (8)	0.0245 (8)
N32	0.0485 (11)	0.0443 (10)	0.0612 (11)	0.0080 (8)	0.0232 (9)	0.0291 (9)
C33	0.0492 (12)	0.0416 (11)	0.0514 (12)	0.0091 (9)	0.0252 (10)	0.0228 (9)
C34	0.0422 (11)	0.0403 (10)	0.0463 (11)	0.0090 (8)	0.0219 (9)	0.0191 (9)
C35	0.0463 (11)	0.0375 (10)	0.0512 (11)	0.0100 (9)	0.0256 (9)	0.0212 (9)
C311	0.0418 (11)	0.0519 (12)	0.0499 (11)	0.0116 (9)	0.0236 (9)	0.0246 (10)
C312	0.0542 (14)	0.0539 (14)	0.0674 (15)	0.0149 (11)	0.0269 (12)	0.0235 (12)
C313	0.0613 (16)	0.0702 (17)	0.0894 (19)	0.0309 (13)	0.0369 (15)	0.0399 (15)
C314	0.0505 (14)	0.090 (2)	0.0758 (17)	0.0230 (14)	0.0233 (13)	0.0408 (16)
C315	0.0518 (14)	0.0762 (18)	0.0609 (15)	0.0108 (13)	0.0165 (12)	0.0197 (13)
C316	0.0495 (13)	0.0558 (13)	0.0584 (13)	0.0142 (10)	0.0248 (11)	0.0193 (11)
C331	0.0663 (15)	0.0588 (14)	0.0765 (16)	0.0175 (12)	0.0304 (13)	0.0418 (13)
O351	0.0583 (9)	0.0427 (8)	0.0694 (10)	0.0185 (7)	0.0374 (8)	0.0310 (7)
C351	0.0370 (10)	0.0452 (11)	0.0485 (11)	0.0107 (8)	0.0155 (9)	0.0269 (9)
C352	0.0491 (12)	0.0476 (12)	0.0575 (13)	0.0125 (10)	0.0163 (10)	0.0307 (10)
C353	0.0742 (17)	0.0632 (16)	0.0749 (17)	0.0127 (13)	0.0264 (14)	0.0464 (14)
C354	0.089 (2)	0.091 (2)	0.0798 (18)	0.0248 (16)	0.0440 (16)	0.0589 (17)
C355	0.0818 (18)	0.0828 (18)	0.0608 (15)	0.0339 (15)	0.0380 (14)	0.0411 (14)
C356	0.0580 (13)	0.0497 (12)	0.0547 (13)	0.0166 (10)	0.0219 (11)	0.0267 (11)
C357	0.104 (2)	0.0467 (14)	0.0809 (18)	0.0248 (14)	0.0384 (16)	0.0318 (13)

### 1-(4-Azidophenyl)-3-[3-methyl-5-(2-methylphenoxy)-1-phenyl-1H-pyrazol-4-yl]prop-2-en-1-one (IIa) Geometric parameters (Å, º)

**Table:**

C1—O1	1.226 (3)	C311—C316	1.380 (3)
C1—C2	1.468 (3)	C312—C313	1.386 (3)
C1—C11	1.488 (3)	C312—H312	0.9300
C2—C3	1.334 (3)	C313—C314	1.375 (4)
C2—H2	0.9300	C313—H313	0.9300
C3—C34	1.440 (3)	C314—C315	1.373 (4)
C3—H3	0.9300	C314—H314	0.9300
C11—C16	1.389 (3)	C315—C316	1.379 (3)
C11—C12	1.392 (3)	C315—H315	0.9300
C12—C13	1.382 (3)	C316—H316	0.9300
C12—H12	0.9300	C331—H33A	0.9600
C13—C14	1.380 (3)	C331—H33B	0.9600
C13—H13	0.9300	C331—H33C	0.9600
C14—C15	1.384 (3)	O351—C351	1.405 (2)
C14—N14	1.422 (3)	C351—C356	1.377 (3)
C15—C16	1.383 (3)	C351—C352	1.391 (3)
C15—H15	0.9300	C352—C353	1.389 (3)
C16—H16	0.9300	C352—C357	1.493 (3)
N14—N15	1.245 (3)	C353—C354	1.372 (4)
N15—N16	1.124 (3)	C353—H353	0.9300
N31—C35	1.351 (3)	C354—C355	1.377 (4)
N31—N32	1.378 (2)	C354—H354	0.9300
N31—C311	1.433 (3)	C355—C356	1.385 (3)
N32—C33	1.326 (3)	C355—H355	0.9300
C33—C34	1.426 (3)	C356—H356	0.9300
C33—C331	1.493 (3)	C357—H35A	0.9600
C34—C35	1.382 (3)	C357—H35B	0.9600
C35—O351	1.358 (2)	C357—H35C	0.9600
C311—C312	1.379 (3)		
			
O1—C1—C2	120.91 (19)	C311—C312—H312	120.5
O1—C1—C11	119.34 (19)	C313—C312—H312	120.5
C2—C1—C11	119.74 (18)	C314—C313—C312	120.5 (2)
C3—C2—C1	120.4 (2)	C314—C313—H313	119.8
C3—C2—H2	119.8	C312—C313—H313	119.8
C1—C2—H2	119.8	C315—C314—C313	120.1 (2)
C2—C3—C34	129.2 (2)	C315—C314—H314	119.9
C2—C3—H3	115.4	C313—C314—H314	119.9
C34—C3—H3	115.4	C314—C315—C316	120.0 (2)
C16—C11—C12	118.03 (19)	C314—C315—H315	120.0
C16—C11—C1	124.29 (18)	C316—C315—H315	120.0
C12—C11—C1	117.66 (18)	C315—C316—C311	119.9 (2)
C13—C12—C11	121.75 (19)	C315—C316—H316	120.1
C13—C12—H12	119.1	C311—C316—H316	120.1
C11—C12—H12	119.1	C33—C331—H33A	109.5
C14—C13—C12	119.10 (19)	C33—C331—H33B	109.5
C14—C13—H13	120.5	H33A—C331—H33B	109.5
C12—C13—H13	120.5	C33—C331—H33C	109.5
C13—C14—C15	120.35 (19)	H33A—C331—H33C	109.5
C13—C14—N14	123.5 (2)	H33B—C331—H33C	109.5
C15—C14—N14	116.1 (2)	C35—O351—C351	119.16 (15)
C16—C15—C14	120.0 (2)	C356—C351—C352	123.0 (2)
C16—C15—H15	120.0	C356—C351—O351	121.83 (18)
C14—C15—H15	120.0	C352—C351—O351	115.11 (18)
C15—C16—C11	120.80 (19)	C353—C352—C351	116.3 (2)
C15—C16—H16	119.6	C353—C352—C357	122.0 (2)
C11—C16—H16	119.6	C351—C352—C357	121.7 (2)
N15—N14—C14	116.1 (2)	C354—C353—C352	121.9 (2)
N16—N15—N14	171.8 (3)	C354—C353—H353	119.1
C35—N31—N32	111.11 (17)	C352—C353—H353	119.1
C35—N31—C311	129.06 (18)	C353—C354—C355	120.3 (2)
N32—N31—C311	119.38 (16)	C353—C354—H354	119.8
C33—N32—N31	104.82 (16)	C355—C354—H354	119.8
N32—C33—C34	112.21 (18)	C354—C355—C356	119.8 (3)
N32—C33—C331	120.41 (18)	C354—C355—H355	120.1
C34—C33—C331	127.4 (2)	C356—C355—H355	120.1
C35—C34—C33	103.40 (17)	C351—C356—C355	118.7 (2)
C35—C34—C3	131.01 (19)	C351—C356—H356	120.7
C33—C34—C3	125.58 (19)	C355—C356—H356	120.7
N31—C35—O351	119.52 (18)	C352—C357—H35A	109.5
N31—C35—C34	108.45 (18)	C352—C357—H35B	109.5
O351—C35—C34	131.82 (18)	H35A—C357—H35B	109.5
C312—C311—C316	120.5 (2)	C352—C357—H35C	109.5
C312—C311—N31	121.14 (19)	H35A—C357—H35C	109.5
C316—C311—N31	118.35 (19)	H35B—C357—H35C	109.5
C311—C312—C313	119.0 (2)		
			
O1—C1—C2—C3	−0.1 (3)	C311—N31—C35—C34	−171.57 (19)
C11—C1—C2—C3	178.86 (19)	C33—C34—C35—N31	−0.9 (2)
C1—C2—C3—C34	−176.53 (19)	C3—C34—C35—N31	178.1 (2)
O1—C1—C11—C16	164.2 (2)	C33—C34—C35—O351	173.7 (2)
C2—C1—C11—C16	−14.8 (3)	C3—C34—C35—O351	−7.3 (4)
O1—C1—C11—C12	−14.2 (3)	C35—N31—C311—C312	−53.4 (3)
C2—C1—C11—C12	166.82 (18)	N32—N31—C311—C312	135.1 (2)
C16—C11—C12—C13	−1.0 (3)	C35—N31—C311—C316	127.5 (2)
C1—C11—C12—C13	177.49 (19)	N32—N31—C311—C316	−44.0 (3)
C11—C12—C13—C14	0.4 (3)	C316—C311—C312—C313	−0.9 (4)
C12—C13—C14—C15	0.4 (3)	N31—C311—C312—C313	−180.0 (2)
C12—C13—C14—N14	−179.8 (2)	C311—C312—C313—C314	0.2 (4)
C13—C14—C15—C16	−0.7 (3)	C312—C313—C314—C315	0.4 (4)
N14—C14—C15—C16	179.5 (2)	C313—C314—C315—C316	−0.3 (4)
C14—C15—C16—C11	0.1 (4)	C314—C315—C316—C311	−0.4 (4)
C12—C11—C16—C15	0.7 (3)	C312—C311—C316—C315	1.0 (3)
C1—C11—C16—C15	−177.7 (2)	N31—C311—C316—C315	−179.9 (2)
C13—C14—N14—N15	−2.7 (3)	N31—C35—O351—C351	−115.8 (2)
C15—C14—N14—N15	177.1 (2)	C34—C35—O351—C351	70.1 (3)
C35—N31—N32—C33	0.1 (2)	C35—O351—C351—C356	21.6 (3)
C311—N31—N32—C33	173.08 (17)	C35—O351—C351—C352	−159.61 (18)
N31—N32—C33—C34	−0.7 (2)	C356—C351—C352—C353	−1.0 (3)
N31—N32—C33—C331	177.90 (19)	O351—C351—C352—C353	−179.69 (19)
N32—C33—C34—C35	1.0 (2)	C356—C351—C352—C357	180.0 (2)
C331—C33—C34—C35	−177.5 (2)	O351—C351—C352—C357	1.3 (3)
N32—C33—C34—C3	−178.03 (18)	C351—C352—C353—C354	0.4 (4)
C331—C33—C34—C3	3.5 (3)	C357—C352—C353—C354	179.4 (3)
C2—C3—C34—C35	0.2 (4)	C352—C353—C354—C355	0.3 (4)
C2—C3—C34—C33	179.0 (2)	C353—C354—C355—C356	−0.4 (4)
N32—N31—C35—O351	−174.87 (17)	C352—C351—C356—C355	0.8 (3)
C311—N31—C35—O351	13.1 (3)	O351—C351—C356—C355	179.5 (2)
N32—N31—C35—C34	0.5 (2)	C354—C355—C356—C351	−0.1 (4)

### 1-(4-Azidophenyl)-3-[3-methyl-5-(2-methylphenoxy)-1-phenyl-1H-pyrazol-4-yl]prop-2-en-1-one (IIa) Hydrogen-bond geometry (Å, º)

**Table:**

*D*—H···*A*	*D*—H	H···*A*	*D*···*A*	*D*—H···*A*
C357—H35*B*···O1^i^	0.96	2.51	3.396 (4)	154

Symmetry code: (i) −*x*+1, −*y*+1, −*z*+2.

### 1-(4-Azidophenyl)-3-[5-(2,4-dichlorophenoxy)-3-methyl-1-phenyl-1H-pyrazol-4-yl]prop-2-en-1-one (IId) Crystal data

**Table:**

C_25_H_17_Cl_2_N_5_O_2_	*F*(000) = 2016
*M**_r_* = 490.33	*D*_x_ = 1.377 Mg m^−^^3^
Monoclinic, *C*2/*c*	Mo *K*α radiation, λ = 0.71073 Å
*a* = 28.1916 (17) Å	Cell parameters from 4176 reflections
*b* = 8.0537 (5) Å	θ = 2.9–25.0°
*c* = 22.0446 (12) Å	µ = 0.31 mm^−^^1^
β = 109.070 (1)°	*T* = 297 K
*V* = 4730.5 (5) Å^3^	Block, colourless
*Z* = 8	0.18 × 0.15 × 0.15 mm

### 1-(4-Azidophenyl)-3-[5-(2,4-dichlorophenoxy)-3-methyl-1-phenyl-1H-pyrazol-4-yl]prop-2-en-1-one (IId) Data collection

**Table:**

Bruker APEXII diffractometer	4174 independent reflections
Radiation source: fine focussealed tube	3181 reflections with *I* > 2σ(*I*)
Graphite monochromator	*R*_int_ = 0.049
φ and ω scans	θ_max_ = 25.0°, θ_min_ = 2.9°
Absorption correction: multi-scan (SADABS; Bruker, 2016)	*h* = −33→33
*T*_min_ = 0.881, *T*_max_ = 0.955	*k* = −9→9
31508 measured reflections	*l* = −26→21

### 1-(4-Azidophenyl)-3-[5-(2,4-dichlorophenoxy)-3-methyl-1-phenyl-1H-pyrazol-4-yl]prop-2-en-1-one (IId) Refinement

**Table:**

Refinement on *F*^2^	Hydrogen site location: inferred from neighbouring sites
Least-squares matrix: full	H-atom parameters constrained
*R*[*F*^2^ > 2σ(*F*^2^)] = 0.086	*w* = 1/[σ^2^(*F*_o_^2^) + (0.0265*P*)^2^ + 10.5248*P*] where *P* = (*F*_o_^2^ + 2*F*_c_^2^)/3
*wR*(*F*^2^) = 0.155	(Δ/σ)_max_ < 0.001
*S* = 1.36	Δρ_max_ = 0.21 e Å^−^^3^
4174 reflections	Δρ_min_ = −0.23 e Å^−^^3^
382 parameters	Extinction correction: SHELXL, Fc^*^=kFc[1+0.001xFc^2^λ^3^/sin(2θ)]^-1/4^
291 restraints	Extinction coefficient: 0.0018 (3)
Primary atom site location: dual	

### 1-(4-Azidophenyl)-3-[5-(2,4-dichlorophenoxy)-3-methyl-1-phenyl-1H-pyrazol-4-yl]prop-2-en-1-one (IId) Special details

**Table:**

Geometry. All esds (except the esd in the dihedral angle between two l.s. planes) are estimated using the full covariance matrix. The cell esds are taken into account individually in the estimation of esds in distances, angles and torsion angles; correlations between esds in cell parameters are only used when they are defined by crystal symmetry. An approximate (isotropic) treatment of cell esds is used for estimating esds involving l.s. planes.

### 1-(4-Azidophenyl)-3-[5-(2,4-dichlorophenoxy)-3-methyl-1-phenyl-1H-pyrazol-4-yl]prop-2-en-1-one (IId) Fractional atomic coordinates and isotropic or equivalent isotropic displacement parameters (Å^2^)

**Table:**

	*x*	*y*	*z*	*U*_iso_*/*U*_eq_	Occ. (<1)
C1	0.42395 (14)	0.7441 (5)	0.5060 (2)	0.0497 (10)	
O1	0.42708 (11)	0.8068 (4)	0.55744 (16)	0.0731 (10)	
C2	0.46893 (14)	0.7129 (5)	0.48791 (19)	0.0509 (10)	
H2	0.4658	0.6524	0.4509	0.061*	
C3	0.51394 (14)	0.7686 (5)	0.52294 (19)	0.0505 (10)	
H3	0.5154	0.8257	0.5602	0.061*	
C11	0.37362 (14)	0.6992 (5)	0.45998 (19)	0.0472 (10)	
C12	0.33110 (15)	0.7460 (6)	0.4740 (2)	0.0637 (12)	
H12	0.3346	0.8036	0.5118	0.076*	
C13	0.28398 (17)	0.7089 (7)	0.4332 (2)	0.0741 (14)	
H13	0.2558	0.7415	0.4433	0.089*	
C14	0.27855 (15)	0.6239 (6)	0.3778 (2)	0.0624 (12)	
C15	0.31991 (16)	0.5748 (6)	0.3626 (2)	0.0665 (13)	
H15	0.3162	0.5164	0.3250	0.080*	
C16	0.36695 (15)	0.6129 (6)	0.4038 (2)	0.0616 (12)	
H16	0.3950	0.5796	0.3935	0.074*	
N14	0.22805 (15)	0.5915 (6)	0.3381 (2)	0.0911 (15)	
N15	0.22323 (16)	0.4999 (7)	0.2916 (3)	0.0912 (15)	
N16	0.2133 (2)	0.4157 (9)	0.2483 (3)	0.128 (2)	
N31	0.61980 (11)	0.6957 (4)	0.46689 (15)	0.0498 (9)	
N32	0.64431 (12)	0.7692 (5)	0.52519 (15)	0.0577 (10)	
C33	0.60890 (15)	0.8028 (5)	0.55055 (19)	0.0539 (11)	
C34	0.56086 (13)	0.7514 (5)	0.51031 (18)	0.0455 (9)	
C35	0.56997 (13)	0.6852 (5)	0.45798 (18)	0.0449 (9)	
C311	0.64806 (14)	0.6459 (5)	0.42690 (18)	0.0489 (10)	
C312	0.63370 (15)	0.5118 (6)	0.3859 (2)	0.0576 (11)	
H312	0.6047	0.4527	0.3832	0.069*	
C313	0.66276 (18)	0.4666 (6)	0.3491 (2)	0.0702 (13)	
H313	0.6530	0.3781	0.3207	0.084*	
C314	0.70616 (19)	0.5516 (7)	0.3541 (2)	0.0782 (15)	
H314	0.7257	0.5199	0.3293	0.094*	
C315	0.72076 (17)	0.6827 (7)	0.3955 (3)	0.0784 (15)	
H315	0.7505	0.7383	0.3993	0.094*	
C316	0.69143 (16)	0.7326 (6)	0.4316 (2)	0.0672 (13)	
H316	0.7008	0.8237	0.4588	0.081*	
C331	0.62281 (17)	0.8821 (7)	0.6150 (2)	0.0720 (14)	
H33A	0.6193	0.8029	0.6457	0.108*	
H33B	0.6011	0.9752	0.6133	0.108*	
H33C	0.6570	0.9194	0.6274	0.108*	
O351	0.53813 (9)	0.6158 (3)	0.40327 (12)	0.0483 (7)	0.55 (4)
C351	0.5093 (7)	0.7253 (10)	0.3559 (10)	0.030 (5)	0.55 (4)
C352	0.4689 (8)	0.6575 (11)	0.3090 (11)	0.038 (5)	0.55 (4)
Cl52	0.4590 (5)	0.4465 (11)	0.3077 (7)	0.118 (4)	0.55 (4)
C353	0.4349 (9)	0.7567 (14)	0.2647 (12)	0.048 (6)	0.55 (4)
H353	0.4104	0.7108	0.2295	0.058*	0.55 (4)
C354	0.4384 (7)	0.9280 (13)	0.2743 (10)	0.039 (5)	0.55 (4)
Cl54	0.3988 (2)	1.0506 (7)	0.2131 (2)	0.067 (3)	0.55 (4)
C355	0.4787 (8)	0.9973 (14)	0.3206 (11)	0.043 (5)	0.55 (4)
H355	0.4832	1.1119	0.3225	0.051*	0.55 (4)
C356	0.5125 (8)	0.8960 (12)	0.3640 (12)	0.047 (6)	0.55 (4)
H356	0.5374	0.9420	0.3987	0.056*	0.55 (4)
O451	0.53813 (9)	0.6158 (3)	0.40327 (12)	0.0483 (7)	0.45 (4)
C451	0.5023 (8)	0.7239 (13)	0.3633 (11)	0.031 (5)	0.45 (4)
C452	0.4619 (10)	0.6533 (14)	0.3172 (14)	0.040 (7)	0.45 (4)
Cl62	0.4539 (3)	0.4410 (9)	0.3133 (5)	0.058 (3)	0.45 (4)
C453	0.4287 (11)	0.7504 (17)	0.2708 (15)	0.047 (6)	0.45 (4)
H453	0.3980	0.7085	0.2450	0.056*	0.45 (4)
C454	0.4429 (9)	0.913 (2)	0.2639 (13)	0.050 (7)	0.45 (4)
Cl64	0.3977 (4)	1.0402 (17)	0.2112 (5)	0.128 (6)	0.45 (4)
C455	0.4832 (9)	0.9854 (19)	0.3095 (14)	0.045 (6)	0.45 (4)
H455	0.4889	1.0989	0.3086	0.054*	0.45 (4)
C456	0.5150 (8)	0.8875 (16)	0.3564 (13)	0.036 (5)	0.45 (4)
H456	0.5450	0.9310	0.3835	0.043*	0.45 (4)

### 1-(4-Azidophenyl)-3-[5-(2,4-dichlorophenoxy)-3-methyl-1-phenyl-1H-pyrazol-4-yl]prop-2-en-1-one (IId) Atomic displacement parameters (Å^2^)

**Table:**

	*U*^11^	*U*^22^	*U*^33^	*U*^12^	*U*^13^	*U*^23^
C1	0.048 (2)	0.045 (2)	0.056 (3)	−0.0079 (19)	0.016 (2)	−0.003 (2)
O1	0.0606 (19)	0.091 (3)	0.072 (2)	−0.0197 (17)	0.0267 (16)	−0.0303 (19)
C2	0.048 (2)	0.056 (3)	0.047 (2)	−0.0035 (19)	0.0118 (19)	−0.003 (2)
C3	0.051 (2)	0.053 (2)	0.045 (2)	−0.004 (2)	0.0127 (19)	−0.0012 (19)
C11	0.048 (2)	0.043 (2)	0.051 (2)	0.0000 (18)	0.0167 (19)	0.0025 (19)
C12	0.051 (3)	0.079 (3)	0.061 (3)	0.006 (2)	0.018 (2)	−0.008 (2)
C13	0.049 (3)	0.100 (4)	0.071 (3)	0.011 (3)	0.017 (2)	−0.004 (3)
C14	0.045 (2)	0.069 (3)	0.065 (3)	−0.001 (2)	0.006 (2)	0.007 (2)
C15	0.051 (3)	0.079 (3)	0.063 (3)	−0.001 (2)	0.011 (2)	−0.016 (3)
C16	0.044 (2)	0.072 (3)	0.070 (3)	−0.002 (2)	0.019 (2)	−0.010 (3)
N14	0.053 (3)	0.113 (4)	0.091 (3)	0.001 (2)	0.003 (2)	−0.017 (3)
N15	0.061 (3)	0.122 (4)	0.077 (3)	−0.009 (3)	0.004 (3)	−0.003 (3)
N16	0.105 (4)	0.175 (6)	0.087 (4)	−0.023 (4)	0.009 (3)	−0.030 (4)
N31	0.0388 (18)	0.061 (2)	0.0452 (19)	−0.0027 (16)	0.0082 (15)	0.0027 (16)
N32	0.0398 (18)	0.081 (3)	0.044 (2)	−0.0093 (18)	0.0023 (15)	0.0002 (18)
C33	0.045 (2)	0.066 (3)	0.046 (2)	−0.008 (2)	0.0093 (19)	0.002 (2)
C34	0.038 (2)	0.049 (2)	0.044 (2)	−0.0037 (18)	0.0057 (17)	0.0053 (19)
C35	0.037 (2)	0.047 (2)	0.044 (2)	−0.0005 (17)	0.0041 (17)	0.0081 (19)
C311	0.038 (2)	0.057 (3)	0.046 (2)	0.0033 (19)	0.0063 (18)	0.012 (2)
C312	0.047 (2)	0.059 (3)	0.065 (3)	0.004 (2)	0.016 (2)	0.010 (2)
C313	0.063 (3)	0.074 (3)	0.072 (3)	0.015 (3)	0.020 (3)	−0.002 (3)
C314	0.065 (3)	0.103 (4)	0.073 (3)	0.016 (3)	0.032 (3)	0.006 (3)
C315	0.052 (3)	0.105 (4)	0.081 (4)	−0.010 (3)	0.025 (3)	0.006 (3)
C316	0.051 (3)	0.083 (3)	0.064 (3)	−0.007 (2)	0.015 (2)	−0.001 (3)
C331	0.057 (3)	0.103 (4)	0.050 (3)	−0.017 (3)	0.010 (2)	−0.012 (3)
O351	0.0413 (14)	0.0446 (15)	0.0480 (16)	−0.0012 (12)	−0.0005 (12)	−0.0026 (12)
C351	0.019 (5)	0.035 (6)	0.043 (8)	−0.004 (4)	0.018 (5)	−0.006 (4)
C352	0.035 (6)	0.041 (6)	0.043 (8)	0.001 (4)	0.017 (7)	−0.008 (5)
Cl52	0.132 (7)	0.064 (4)	0.115 (6)	−0.019 (4)	−0.018 (4)	−0.012 (4)
C353	0.035 (7)	0.067 (9)	0.039 (8)	−0.011 (6)	0.008 (6)	−0.013 (6)
C354	0.042 (6)	0.046 (6)	0.030 (7)	0.028 (5)	0.013 (6)	0.010 (5)
Cl54	0.058 (3)	0.068 (4)	0.051 (3)	0.033 (2)	−0.014 (3)	0.020 (2)
C355	0.046 (6)	0.045 (6)	0.039 (7)	0.009 (5)	0.017 (7)	−0.007 (5)
C356	0.052 (10)	0.049 (8)	0.035 (7)	0.000 (6)	0.010 (7)	−0.016 (6)
O451	0.0413 (14)	0.0446 (15)	0.0480 (16)	−0.0012 (12)	−0.0005 (12)	−0.0026 (12)
C451	0.026 (8)	0.057 (9)	0.018 (5)	0.000 (5)	0.017 (6)	0.000 (5)
C452	0.040 (9)	0.050 (8)	0.037 (8)	−0.011 (6)	0.021 (7)	−0.006 (6)
Cl62	0.052 (4)	0.036 (3)	0.073 (4)	−0.012 (2)	0.001 (2)	−0.013 (2)
C453	0.035 (8)	0.067 (10)	0.039 (8)	0.002 (7)	0.011 (8)	0.000 (7)
C454	0.042 (8)	0.077 (11)	0.036 (9)	0.005 (8)	0.020 (6)	−0.008 (7)
Cl64	0.103 (8)	0.150 (10)	0.130 (9)	0.036 (6)	0.037 (7)	0.023 (6)
C455	0.057 (9)	0.041 (8)	0.047 (10)	0.009 (6)	0.032 (7)	0.003 (7)
C456	0.020 (7)	0.047 (10)	0.043 (10)	−0.009 (7)	0.012 (6)	0.002 (8)

### 1-(4-Azidophenyl)-3-[5-(2,4-dichlorophenoxy)-3-methyl-1-phenyl-1H-pyrazol-4-yl]prop-2-en-1-one (IId) Geometric parameters (Å, º)

**Table:**

C1—O1	1.218 (5)	C313—H313	0.9300
C1—C2	1.470 (5)	C314—C315	1.367 (7)
C1—C11	1.494 (5)	C314—H314	0.9300
C2—C3	1.329 (5)	C315—C316	1.382 (6)
C2—H2	0.9300	C315—H315	0.9300
C3—C34	1.445 (5)	C316—H316	0.9300
C3—H3	0.9300	C331—H33A	0.9600
C11—C16	1.378 (6)	C331—H33B	0.9600
C11—C12	1.385 (5)	C331—H33C	0.9600
C12—C13	1.372 (6)	O351—C351	1.405 (7)
C12—H12	0.9300	C351—C352	1.377 (7)
C13—C14	1.365 (6)	C351—C356	1.385 (11)
C13—H13	0.9300	C352—C353	1.377 (8)
C14—C15	1.372 (6)	C352—Cl52	1.721 (7)
C14—N14	1.431 (6)	C353—C354	1.395 (13)
C15—C16	1.375 (6)	C353—H353	0.9300
C15—H15	0.9300	C354—C355	1.374 (8)
C16—H16	0.9300	C354—Cl54	1.748 (6)
N14—N15	1.233 (7)	C355—C356	1.375 (8)
N15—N16	1.128 (7)	C355—H355	0.9300
N31—C35	1.356 (5)	C356—H356	0.9300
N31—N32	1.378 (4)	C451—C452	1.378 (8)
N31—C311	1.425 (5)	C451—C456	1.387 (12)
N32—C33	1.322 (5)	C452—C453	1.381 (8)
C33—C34	1.417 (5)	C452—Cl62	1.723 (7)
C33—C331	1.488 (6)	C453—C454	1.394 (15)
C34—C35	1.369 (5)	C453—H453	0.9300
C35—O351	1.366 (4)	C454—C455	1.376 (9)
C311—C312	1.381 (6)	C454—Cl64	1.746 (8)
C311—C316	1.382 (6)	C455—C456	1.373 (8)
C312—C313	1.378 (6)	C455—H455	0.9300
C312—H312	0.9300	C456—H456	0.9300
C313—C314	1.374 (7)		
			
O1—C1—C2	121.1 (4)	C315—C314—C313	120.3 (5)
O1—C1—C11	119.8 (4)	C315—C314—H314	119.8
C2—C1—C11	119.1 (4)	C313—C314—H314	119.8
C3—C2—C1	121.9 (4)	C314—C315—C316	120.1 (5)
C3—C2—H2	119.0	C314—C315—H315	119.9
C1—C2—H2	119.0	C316—C315—H315	119.9
C2—C3—C34	128.0 (4)	C315—C316—C311	119.4 (5)
C2—C3—H3	116.0	C315—C316—H316	120.3
C34—C3—H3	116.0	C311—C316—H316	120.3
C16—C11—C12	117.7 (4)	C33—C331—H33A	109.5
C16—C11—C1	123.5 (4)	C33—C331—H33B	109.5
C12—C11—C1	118.8 (4)	H33A—C331—H33B	109.5
C13—C12—C11	121.2 (4)	C33—C331—H33C	109.5
C13—C12—H12	119.4	H33A—C331—H33C	109.5
C11—C12—H12	119.4	H33B—C331—H33C	109.5
C14—C13—C12	119.8 (4)	C35—O351—C351	117.0 (5)
C14—C13—H13	120.1	C352—C351—C356	119.5 (7)
C12—C13—H13	120.1	C352—C351—O351	116.5 (6)
C13—C14—C15	120.5 (4)	C356—C351—O351	122.0 (9)
C13—C14—N14	115.9 (4)	C351—C352—C353	121.1 (6)
C15—C14—N14	123.6 (5)	C351—C352—Cl52	119.6 (6)
C14—C15—C16	119.2 (4)	C353—C352—Cl52	119.3 (6)
C14—C15—H15	120.4	C352—C353—C354	117.9 (8)
C16—C15—H15	120.4	C352—C353—H353	121.1
C15—C16—C11	121.6 (4)	C354—C353—H353	121.1
C15—C16—H16	119.2	C355—C354—C353	121.0 (8)
C11—C16—H16	119.2	C355—C354—Cl54	120.5 (6)
N15—N14—C14	115.8 (5)	C353—C354—Cl54	116.5 (8)
N16—N15—N14	172.3 (6)	C354—C355—C356	119.5 (7)
C35—N31—N32	110.0 (3)	C354—C355—H355	120.3
C35—N31—C311	130.9 (3)	C356—C355—H355	120.3
N32—N31—C311	119.1 (3)	C355—C356—C351	119.9 (8)
C33—N32—N31	105.3 (3)	C355—C356—H356	120.0
N32—C33—C34	112.0 (4)	C351—C356—H356	120.0
N32—C33—C331	119.4 (4)	C452—C451—C456	119.5 (9)
C34—C33—C331	128.5 (4)	C451—C452—C453	120.6 (8)
C35—C34—C33	103.7 (3)	C451—C452—Cl62	120.6 (7)
C35—C34—C3	129.5 (3)	C453—C452—Cl62	118.6 (7)
C33—C34—C3	126.8 (4)	C452—C453—C454	117.7 (11)
N31—C35—O351	120.3 (3)	C452—C453—H453	121.1
N31—C35—C34	108.9 (3)	C454—C453—H453	121.1
O351—C35—C34	130.8 (3)	C455—C454—C453	121.0 (10)
C312—C311—C316	120.5 (4)	C455—C454—Cl64	119.3 (9)
C312—C311—N31	121.4 (4)	C453—C454—Cl64	116.8 (11)
C316—C311—N31	118.1 (4)	C456—C455—C454	119.2 (8)
C313—C312—C311	119.2 (4)	C456—C455—H455	120.4
C313—C312—H312	120.4	C454—C455—H455	120.4
C311—C312—H312	120.4	C455—C456—C451	120.0 (9)
C314—C313—C312	120.4 (5)	C455—C456—H456	120.0
C314—C313—H313	119.8	C451—C456—H456	120.0
C312—C313—H313	119.8		
			
O1—C1—C2—C3	−8.0 (6)	N32—N31—C311—C312	149.6 (4)
C11—C1—C2—C3	171.4 (4)	C35—N31—C311—C316	151.0 (4)
C1—C2—C3—C34	−178.2 (4)	N32—N31—C311—C316	−28.4 (5)
O1—C1—C11—C16	−173.2 (4)	C316—C311—C312—C313	−0.9 (6)
C2—C1—C11—C16	7.4 (6)	N31—C311—C312—C313	−178.8 (4)
O1—C1—C11—C12	6.7 (6)	C311—C312—C313—C314	1.5 (7)
C2—C1—C11—C12	−172.7 (4)	C312—C313—C314—C315	−0.4 (8)
C16—C11—C12—C13	−0.5 (7)	C313—C314—C315—C316	−1.3 (8)
C1—C11—C12—C13	179.6 (4)	C314—C315—C316—C311	1.9 (7)
C11—C12—C13—C14	0.2 (8)	C312—C311—C316—C315	−0.8 (7)
C12—C13—C14—C15	0.2 (8)	N31—C311—C316—C315	177.2 (4)
C12—C13—C14—N14	−179.7 (5)	N31—C35—O351—C351	−102.1 (15)
C13—C14—C15—C16	−0.3 (8)	C34—C35—O351—C351	78.3 (16)
N14—C14—C15—C16	179.6 (5)	C35—O351—C351—C352	−164 (2)
C14—C15—C16—C11	0.0 (7)	C35—O351—C351—C356	0 (4)
C12—C11—C16—C15	0.4 (7)	C356—C351—C352—C353	8 (5)
C1—C11—C16—C15	−179.7 (4)	O351—C351—C352—C353	172 (3)
C13—C14—N14—N15	−172.6 (5)	C356—C351—C352—Cl52	−169 (3)
C15—C14—N14—N15	7.5 (8)	O351—C351—C352—Cl52	−4 (4)
C35—N31—N32—C33	−0.3 (4)	C351—C352—C353—C354	−9 (5)
C311—N31—N32—C33	179.3 (4)	Cl52—C352—C353—C354	168 (3)
N31—N32—C33—C34	0.6 (5)	C352—C353—C354—C355	10 (5)
N31—N32—C33—C331	179.2 (4)	C352—C353—C354—Cl54	174 (3)
N32—C33—C34—C35	−0.6 (5)	C353—C354—C355—C356	−9 (5)
C331—C33—C34—C35	−179.1 (5)	Cl54—C354—C355—C356	−173 (3)
N32—C33—C34—C3	180.0 (4)	C354—C355—C356—C351	8 (5)
C331—C33—C34—C3	1.5 (7)	C352—C351—C356—C355	−8 (5)
C2—C3—C34—C35	5.1 (7)	O351—C351—C356—C355	−171 (3)
C2—C3—C34—C33	−175.7 (4)	C456—C451—C452—C453	−11 (6)
N32—N31—C35—O351	−179.8 (3)	C456—C451—C452—Cl62	164 (3)
C311—N31—C35—O351	0.7 (6)	C451—C452—C453—C454	13 (6)
N32—N31—C35—C34	−0.1 (5)	Cl62—C452—C453—C454	−163 (4)
C311—N31—C35—C34	−179.6 (4)	C452—C453—C454—C455	−13 (6)
C33—C34—C35—N31	0.4 (4)	C452—C453—C454—Cl64	−173 (3)
C3—C34—C35—N31	179.8 (4)	C453—C454—C455—C456	11 (6)
C33—C34—C35—O351	−179.9 (4)	Cl64—C454—C455—C456	171 (3)
C3—C34—C35—O351	−0.5 (7)	C454—C455—C456—C451	−10 (6)
C35—N31—C311—C312	−31.0 (6)	C452—C451—C456—C455	10 (6)

### 1-(4-Azidophenyl)-3-[5-(2,4-dichlorophenoxy)-3-methyl-1-phenyl-1H-pyrazol-4-yl]prop-2-en-1-one (IId) Hydrogen-bond geometry (Å, º)

**Table:**

*D*—H···*A*	*D*—H	H···*A*	*D*···*A*	*D*—H···*A*
C356—H356···O1^i^	0.93	2.32	3.115 (18)	143
C456—H456···O1^i^	0.93	2.47	3.21 (2)	137

Symmetry code: (i) −*x*+1, −*y*+2, −*z*+1.

### 1-(4-Azidophenyl)-3-[3-methyl-5-(naphthalen-2-yloxy)-1-phenyl-1H-pyrazol-4-yl]prop-2-en-1-on (IIe) Crystal data

**Table:**

C_29_H_21_N_5_O_2_	*F*(000) = 984
*M**_r_* = 471.51	*D*_x_ = 1.324 Mg m^−^^3^
Monoclinic, *P*2_1_/*n*	Mo *K*α radiation, λ = 0.71073 Å
*a* = 9.8460 (8) Å	Cell parameters from 4197 reflections
*b* = 22.4303 (18) Å	θ = 3.1–25.1°
*c* = 11.0490 (9) Å	µ = 0.09 mm^−^^1^
β = 104.157 (2)°	*T* = 297 K
*V* = 2366.0 (3) Å^3^	Block, orange
*Z* = 4	0.22 × 0.21 × 0.16 mm

### 1-(4-Azidophenyl)-3-[3-methyl-5-(naphthalen-2-yloxy)-1-phenyl-1H-pyrazol-4-yl]prop-2-en-1-on (IIe) Data collection

**Table:**

Bruker APEXII diffractometer	4196 independent reflections
Radiation source: fine focussealed tube	2463 reflections with *I* > 2σ(*I*)
Graphite monochromator	*R*_int_ = 0.092
φ and ω scans	θ_max_ = 25.1°, θ_min_ = 3.1°
Absorption correction: multi-scan (SADABS; Bruker, 2016)	*h* = −11→11
*T*_min_ = 0.930, *T*_max_ = 0.986	*k* = −26→26
43217 measured reflections	*l* = −12→13

### 1-(4-Azidophenyl)-3-[3-methyl-5-(naphthalen-2-yloxy)-1-phenyl-1H-pyrazol-4-yl]prop-2-en-1-on (IIe) Refinement

**Table:**

Refinement on *F*^2^	Hydrogen site location: inferred from neighbouring sites
Least-squares matrix: full	H-atom parameters constrained
*R*[*F*^2^ > 2σ(*F*^2^)] = 0.063	*w* = 1/[σ^2^(*F*_o_^2^) + (0.0268*P*)^2^ + 3.4386*P*] where *P* = (*F*_o_^2^ + 2*F*_c_^2^)/3
*wR*(*F*^2^) = 0.154	(Δ/σ)_max_ < 0.001
*S* = 1.06	Δρ_max_ = 0.23 e Å^−^^3^
4196 reflections	Δρ_min_ = −0.27 e Å^−^^3^
327 parameters	Extinction correction: SHELXL, Fc^*^=kFc[1+0.001xFc^2^λ^3^/sin(2θ)]^-1/4^
0 restraints	Extinction coefficient: 0.0143 (9)
Primary atom site location: dual	

### 1-(4-Azidophenyl)-3-[3-methyl-5-(naphthalen-2-yloxy)-1-phenyl-1H-pyrazol-4-yl]prop-2-en-1-on (IIe) Special details

**Table:**

Geometry. All esds (except the esd in the dihedral angle between two l.s. planes) are estimated using the full covariance matrix. The cell esds are taken into account individually in the estimation of esds in distances, angles and torsion angles; correlations between esds in cell parameters are only used when they are defined by crystal symmetry. An approximate (isotropic) treatment of cell esds is used for estimating esds involving l.s. planes.

### 1-(4-Azidophenyl)-3-[3-methyl-5-(naphthalen-2-yloxy)-1-phenyl-1H-pyrazol-4-yl]prop-2-en-1-on (IIe) Fractional atomic coordinates and isotropic or equivalent isotropic displacement parameters (Å^2^)

**Table:**

	*x*	*y*	*z*	*U*_iso_*/*U*_eq_	
C1	0.3799 (4)	0.50783 (16)	0.2247 (3)	0.0478 (9)	
O1	0.2646 (3)	0.49934 (13)	0.1526 (2)	0.0673 (8)	
C2	0.5060 (4)	0.47779 (16)	0.2070 (3)	0.0489 (9)	
H2	0.5904	0.4830	0.2662	0.059*	
C3	0.5026 (4)	0.44316 (15)	0.1078 (3)	0.0465 (9)	
H3	0.4164	0.4406	0.0502	0.056*	
C11	0.3908 (3)	0.54796 (15)	0.3332 (3)	0.0433 (8)	
C12	0.2693 (4)	0.56999 (17)	0.3595 (4)	0.0543 (10)	
H12	0.1831	0.5588	0.3088	0.065*	
C13	0.2725 (4)	0.60784 (17)	0.4580 (4)	0.0552 (10)	
H13	0.1896	0.6212	0.4748	0.066*	
C14	0.3990 (4)	0.62554 (16)	0.5309 (3)	0.0486 (9)	
C15	0.5220 (4)	0.6051 (2)	0.5074 (4)	0.0656 (12)	
H15	0.6078	0.6174	0.5572	0.079*	
C16	0.5169 (4)	0.56643 (19)	0.4095 (4)	0.0595 (11)	
H16	0.6001	0.5524	0.3944	0.071*	
N14	0.4149 (4)	0.66594 (16)	0.6335 (3)	0.0655 (10)	
N15	0.3037 (4)	0.68821 (16)	0.6481 (3)	0.0621 (9)	
N16	0.2126 (4)	0.71190 (18)	0.6714 (4)	0.0877 (13)	
N31	0.8165 (3)	0.36482 (13)	0.0799 (3)	0.0495 (8)	
N32	0.7208 (3)	0.33982 (14)	−0.0191 (3)	0.0551 (8)	
C33	0.6012 (4)	0.36732 (16)	−0.0196 (3)	0.0487 (9)	
C34	0.6151 (4)	0.40921 (15)	0.0783 (3)	0.0444 (9)	
C35	0.7544 (4)	0.40565 (15)	0.1390 (3)	0.0446 (9)	
C311	0.9557 (4)	0.34178 (17)	0.1152 (3)	0.0491 (9)	
C312	1.0685 (4)	0.37933 (18)	0.1521 (3)	0.0530 (10)	
H312	1.0555	0.4204	0.1536	0.064*	
C313	1.2010 (4)	0.3553 (2)	0.1870 (4)	0.0634 (11)	
H313	1.2778	0.3805	0.2116	0.076*	
C314	1.2215 (5)	0.2949 (2)	0.1861 (4)	0.0762 (13)	
H314	1.3115	0.2792	0.2102	0.091*	
C315	1.1087 (5)	0.2577 (2)	0.1496 (5)	0.0831 (15)	
H315	1.1227	0.2167	0.1496	0.100*	
C316	0.9736 (4)	0.28021 (19)	0.1125 (4)	0.0704 (13)	
H316	0.8971	0.2549	0.0866	0.084*	
C331	0.4703 (4)	0.35114 (19)	−0.1150 (4)	0.0665 (12)	
H33A	0.4924	0.3233	−0.1734	0.100*	
H33B	0.4298	0.3864	−0.1585	0.100*	
H33C	0.4047	0.3333	−0.0743	0.100*	
O351	0.8283 (2)	0.42967 (10)	0.2482 (2)	0.0485 (6)	
C351	0.8655 (3)	0.51795 (16)	0.3666 (3)	0.0455 (9)	
H351	0.8589	0.4953	0.4355	0.055*	
C352	0.8523 (3)	0.49169 (15)	0.2527 (3)	0.0423 (8)	
C353	0.8651 (4)	0.52368 (17)	0.1473 (3)	0.0498 (9)	
H353	0.8558	0.5046	0.0709	0.060*	
C354	0.8917 (4)	0.58332 (18)	0.1585 (3)	0.0551 (10)	
H354	0.9021	0.6047	0.0893	0.066*	
C355	0.9276 (4)	0.67581 (18)	0.2850 (4)	0.0664 (12)	
H355	0.9410	0.6977	0.2175	0.080*	
C356	0.9309 (5)	0.7037 (2)	0.3951 (5)	0.0732 (13)	
H356	0.9446	0.7447	0.4017	0.088*	
C357	0.9139 (4)	0.6714 (2)	0.4975 (4)	0.0685 (12)	
H357	0.9152	0.6913	0.5716	0.082*	
C358	0.8951 (4)	0.61082 (19)	0.4919 (4)	0.0570 (10)	
H358	0.8862	0.5897	0.5620	0.068*	
C359	0.9037 (4)	0.61317 (16)	0.2727 (3)	0.0498 (9)	
C360	0.8896 (3)	0.58065 (16)	0.3779 (3)	0.0437 (9)	

### 1-(4-Azidophenyl)-3-[3-methyl-5-(naphthalen-2-yloxy)-1-phenyl-1H-pyrazol-4-yl]prop-2-en-1-on (IIe) Atomic displacement parameters (Å^2^)

**Table:**

	*U*^11^	*U*^22^	*U*^33^	*U*^12^	*U*^13^	*U*^23^
C1	0.042 (2)	0.052 (2)	0.048 (2)	0.0016 (18)	0.0093 (17)	0.0008 (18)
O1	0.0452 (16)	0.089 (2)	0.0630 (17)	0.0038 (15)	0.0042 (14)	−0.0216 (16)
C2	0.037 (2)	0.058 (2)	0.050 (2)	0.0027 (17)	0.0077 (16)	−0.0056 (19)
C3	0.041 (2)	0.051 (2)	0.047 (2)	0.0013 (17)	0.0080 (16)	0.0021 (18)
C11	0.0349 (19)	0.048 (2)	0.046 (2)	0.0009 (16)	0.0098 (16)	−0.0019 (17)
C12	0.036 (2)	0.064 (3)	0.062 (2)	−0.0024 (18)	0.0092 (18)	−0.011 (2)
C13	0.042 (2)	0.063 (3)	0.061 (2)	−0.0017 (19)	0.0130 (18)	−0.014 (2)
C14	0.048 (2)	0.051 (2)	0.045 (2)	−0.0011 (18)	0.0082 (17)	−0.0036 (18)
C15	0.037 (2)	0.093 (3)	0.061 (3)	0.002 (2)	0.0013 (19)	−0.019 (2)
C16	0.039 (2)	0.082 (3)	0.056 (2)	0.007 (2)	0.0096 (18)	−0.013 (2)
N14	0.054 (2)	0.078 (2)	0.064 (2)	0.0027 (19)	0.0129 (17)	−0.0171 (19)
N15	0.066 (2)	0.058 (2)	0.061 (2)	−0.0092 (19)	0.0140 (19)	−0.0108 (18)
N16	0.076 (3)	0.085 (3)	0.107 (3)	0.002 (2)	0.032 (2)	−0.036 (2)
N31	0.0454 (18)	0.0521 (19)	0.0493 (18)	0.0013 (15)	0.0080 (15)	−0.0150 (15)
N32	0.0519 (19)	0.057 (2)	0.0527 (19)	−0.0057 (16)	0.0061 (15)	−0.0154 (16)
C33	0.048 (2)	0.049 (2)	0.047 (2)	−0.0067 (18)	0.0065 (17)	−0.0048 (18)
C34	0.045 (2)	0.045 (2)	0.042 (2)	−0.0008 (17)	0.0099 (16)	−0.0052 (17)
C35	0.046 (2)	0.044 (2)	0.042 (2)	−0.0012 (17)	0.0079 (17)	−0.0064 (17)
C311	0.046 (2)	0.056 (2)	0.047 (2)	0.0025 (19)	0.0137 (17)	−0.0074 (19)
C312	0.050 (2)	0.056 (2)	0.053 (2)	0.000 (2)	0.0148 (18)	−0.0016 (19)
C313	0.048 (2)	0.084 (3)	0.058 (3)	0.001 (2)	0.0133 (19)	−0.006 (2)
C314	0.054 (3)	0.089 (4)	0.086 (3)	0.017 (3)	0.018 (2)	−0.013 (3)
C315	0.072 (3)	0.069 (3)	0.109 (4)	0.019 (3)	0.024 (3)	−0.021 (3)
C316	0.062 (3)	0.059 (3)	0.093 (3)	0.003 (2)	0.023 (2)	−0.023 (2)
C331	0.060 (3)	0.068 (3)	0.066 (3)	−0.009 (2)	0.004 (2)	−0.014 (2)
O351	0.0554 (15)	0.0442 (14)	0.0417 (14)	−0.0010 (12)	0.0042 (11)	−0.0085 (12)
C351	0.040 (2)	0.053 (2)	0.040 (2)	−0.0020 (17)	0.0057 (16)	−0.0060 (18)
C352	0.0357 (19)	0.043 (2)	0.044 (2)	−0.0003 (16)	0.0026 (15)	−0.0079 (18)
C353	0.052 (2)	0.056 (3)	0.040 (2)	0.0036 (18)	0.0078 (17)	−0.0031 (18)
C354	0.058 (2)	0.060 (3)	0.045 (2)	0.005 (2)	0.0081 (18)	0.005 (2)
C355	0.070 (3)	0.053 (3)	0.071 (3)	0.002 (2)	0.007 (2)	−0.001 (2)
C356	0.074 (3)	0.055 (3)	0.083 (3)	0.006 (2)	0.005 (3)	−0.014 (3)
C357	0.056 (3)	0.072 (3)	0.072 (3)	0.000 (2)	0.007 (2)	−0.029 (3)
C358	0.050 (2)	0.070 (3)	0.049 (2)	−0.003 (2)	0.0093 (18)	−0.018 (2)
C359	0.045 (2)	0.052 (2)	0.047 (2)	0.0021 (18)	0.0028 (17)	−0.0025 (19)
C360	0.0306 (18)	0.055 (2)	0.042 (2)	0.0002 (16)	0.0034 (15)	−0.0121 (18)

### 1-(4-Azidophenyl)-3-[3-methyl-5-(naphthalen-2-yloxy)-1-phenyl-1H-pyrazol-4-yl]prop-2-en-1-on (IIe) Geometric parameters (Å, º)

**Table:**

C1—O1	1.232 (4)	C312—H312	0.9300
C1—C2	1.467 (5)	C313—C314	1.371 (6)
C1—C11	1.482 (5)	C313—H313	0.9300
C2—C3	1.337 (5)	C314—C315	1.368 (6)
C2—H2	0.9300	C314—H314	0.9300
C3—C34	1.446 (5)	C315—C316	1.387 (6)
C3—H3	0.9300	C315—H315	0.9300
C11—C16	1.382 (5)	C316—H316	0.9300
C11—C12	1.389 (5)	C331—H33A	0.9600
C12—C13	1.375 (5)	C331—H33B	0.9600
C12—H12	0.9300	C331—H33C	0.9600
C13—C14	1.367 (5)	O351—C352	1.410 (4)
C13—H13	0.9300	C351—C352	1.367 (5)
C14—C15	1.377 (5)	C351—C360	1.427 (5)
C14—N14	1.430 (5)	C351—H351	0.9300
C15—C16	1.378 (5)	C352—C353	1.400 (5)
C15—H15	0.9300	C353—C354	1.363 (5)
C16—H16	0.9300	C353—H353	0.9300
N14—N15	1.249 (5)	C354—C359	1.407 (5)
N15—N16	1.126 (5)	C354—H354	0.9300
N31—C35	1.353 (4)	C355—C356	1.360 (6)
N31—N32	1.376 (4)	C355—C359	1.426 (5)
N31—C311	1.427 (4)	C355—H355	0.9300
N32—C33	1.327 (4)	C356—C357	1.388 (6)
C33—C34	1.414 (5)	C356—H356	0.9300
C33—C331	1.497 (5)	C357—C358	1.371 (6)
C34—C35	1.374 (5)	C357—H357	0.9300
C35—O351	1.359 (4)	C358—C360	1.419 (5)
C311—C312	1.374 (5)	C358—H358	0.9300
C311—C316	1.394 (5)	C359—C360	1.408 (5)
C312—C313	1.377 (5)		
			
O1—C1—C2	121.3 (3)	C314—C313—H313	119.5
O1—C1—C11	119.3 (3)	C312—C313—H313	119.5
C2—C1—C11	119.4 (3)	C315—C314—C313	119.6 (4)
C3—C2—C1	121.6 (3)	C315—C314—H314	120.2
C3—C2—H2	119.2	C313—C314—H314	120.2
C1—C2—H2	119.2	C314—C315—C316	121.0 (4)
C2—C3—C34	128.6 (3)	C314—C315—H315	119.5
C2—C3—H3	115.7	C316—C315—H315	119.5
C34—C3—H3	115.7	C315—C316—C311	118.2 (4)
C16—C11—C12	117.2 (3)	C315—C316—H316	120.9
C16—C11—C1	123.5 (3)	C311—C316—H316	120.9
C12—C11—C1	119.3 (3)	C33—C331—H33A	109.5
C13—C12—C11	122.1 (3)	C33—C331—H33B	109.5
C13—C12—H12	119.0	H33A—C331—H33B	109.5
C11—C12—H12	119.0	C33—C331—H33C	109.5
C14—C13—C12	119.2 (3)	H33A—C331—H33C	109.5
C14—C13—H13	120.4	H33B—C331—H33C	109.5
C12—C13—H13	120.4	C35—O351—C352	118.1 (3)
C13—C14—C15	120.6 (4)	C352—C351—C360	118.8 (3)
C13—C14—N14	124.0 (3)	C352—C351—H351	120.6
C15—C14—N14	115.5 (3)	C360—C351—H351	120.6
C14—C15—C16	119.5 (3)	C351—C352—C353	122.5 (3)
C14—C15—H15	120.2	C351—C352—O351	115.8 (3)
C16—C15—H15	120.2	C353—C352—O351	121.7 (3)
C15—C16—C11	121.5 (3)	C354—C353—C352	118.8 (3)
C15—C16—H16	119.3	C354—C353—H353	120.6
C11—C16—H16	119.3	C352—C353—H353	120.6
N15—N14—C14	115.2 (3)	C353—C354—C359	121.4 (4)
N16—N15—N14	172.4 (4)	C353—C354—H354	119.3
C35—N31—N32	110.9 (3)	C359—C354—H354	119.3
C35—N31—C311	129.0 (3)	C356—C355—C359	120.4 (4)
N32—N31—C311	119.6 (3)	C356—C355—H355	119.8
C33—N32—N31	104.3 (3)	C359—C355—H355	119.8
N32—C33—C34	112.7 (3)	C355—C356—C357	120.5 (4)
N32—C33—C331	120.0 (3)	C355—C356—H356	119.7
C34—C33—C331	127.2 (3)	C357—C356—H356	119.7
C35—C34—C33	103.4 (3)	C358—C357—C356	121.5 (4)
C35—C34—C3	130.5 (3)	C358—C357—H357	119.3
C33—C34—C3	126.0 (3)	C356—C357—H357	119.3
N31—C35—O351	119.3 (3)	C357—C358—C360	119.2 (4)
N31—C35—C34	108.6 (3)	C357—C358—H358	120.4
O351—C35—C34	131.6 (3)	C360—C358—H358	120.4
C312—C311—C316	121.1 (4)	C354—C359—C360	119.4 (3)
C312—C311—N31	120.8 (3)	C354—C359—C355	122.0 (4)
C316—C311—N31	118.1 (3)	C360—C359—C355	118.6 (4)
C311—C312—C313	119.0 (4)	C359—C360—C358	119.7 (3)
C311—C312—H312	120.5	C359—C360—C351	119.1 (3)
C313—C312—H312	120.5	C358—C360—C351	121.2 (4)
C314—C313—C312	121.1 (4)		
			
O1—C1—C2—C3	4.4 (6)	C35—N31—C311—C312	−47.2 (5)
C11—C1—C2—C3	−177.2 (3)	N32—N31—C311—C312	140.9 (3)
C1—C2—C3—C34	−178.0 (3)	C35—N31—C311—C316	132.0 (4)
O1—C1—C11—C16	−171.1 (4)	N32—N31—C311—C316	−39.9 (5)
C2—C1—C11—C16	10.4 (5)	C316—C311—C312—C313	0.0 (6)
O1—C1—C11—C12	7.1 (5)	N31—C311—C312—C313	179.2 (3)
C2—C1—C11—C12	−171.4 (3)	C311—C312—C313—C314	−0.5 (6)
C16—C11—C12—C13	−1.0 (6)	C312—C313—C314—C315	0.3 (7)
C1—C11—C12—C13	−179.3 (4)	C313—C314—C315—C316	0.4 (7)
C11—C12—C13—C14	1.5 (6)	C314—C315—C316—C311	−0.9 (7)
C12—C13—C14—C15	−0.9 (6)	C312—C311—C316—C315	0.7 (6)
C12—C13—C14—N14	178.3 (4)	N31—C311—C316—C315	−178.5 (4)
C13—C14—C15—C16	−0.1 (6)	N31—C35—O351—C352	118.1 (3)
N14—C14—C15—C16	−179.4 (4)	C34—C35—O351—C352	−71.1 (5)
C14—C15—C16—C11	0.6 (7)	C360—C351—C352—C353	1.7 (5)
C12—C11—C16—C15	−0.1 (6)	C360—C351—C352—O351	−179.8 (3)
C1—C11—C16—C15	178.2 (4)	C35—O351—C352—C351	150.9 (3)
C13—C14—N14—N15	−5.0 (6)	C35—O351—C352—C353	−30.7 (4)
C15—C14—N14—N15	174.2 (4)	C351—C352—C353—C354	−0.1 (5)
C35—N31—N32—C33	1.2 (4)	O351—C352—C353—C354	−178.4 (3)
C311—N31—N32—C33	174.5 (3)	C352—C353—C354—C359	−1.2 (5)
N31—N32—C33—C34	−1.1 (4)	C359—C355—C356—C357	1.3 (6)
N31—N32—C33—C331	−179.2 (3)	C355—C356—C357—C358	0.8 (7)
N32—C33—C34—C35	0.5 (4)	C356—C357—C358—C360	−1.6 (6)
C331—C33—C34—C35	178.6 (4)	C353—C354—C359—C360	0.7 (5)
N32—C33—C34—C3	−175.5 (3)	C353—C354—C359—C355	−178.2 (4)
C331—C33—C34—C3	2.5 (6)	C356—C355—C359—C354	176.3 (4)
C2—C3—C34—C35	−3.7 (7)	C356—C355—C359—C360	−2.6 (6)
C2—C3—C34—C33	171.2 (4)	C354—C359—C360—C358	−177.1 (3)
N32—N31—C35—O351	171.8 (3)	C355—C359—C360—C358	1.8 (5)
C311—N31—C35—O351	−0.6 (6)	C354—C359—C360—C351	0.9 (5)
N32—N31—C35—C34	−0.9 (4)	C355—C359—C360—C351	179.9 (3)
C311—N31—C35—C34	−173.4 (3)	C357—C358—C360—C359	0.2 (5)
C33—C34—C35—N31	0.2 (4)	C357—C358—C360—C351	−177.8 (3)
C3—C34—C35—N31	176.0 (3)	C352—C351—C360—C359	−2.1 (5)
C33—C34—C35—O351	−171.3 (4)	C352—C351—C360—C358	175.9 (3)
C3—C34—C35—O351	4.4 (7)		

### 1-(4-Azidophenyl)-3-[3-methyl-5-(naphthalen-2-yloxy)-1-phenyl-1H-pyrazol-4-yl]prop-2-en-1-on (IIe) Hydrogen-bond geometry (Å, º)

**Table:**

*D*—H···*A*	*D*—H	H···*A*	*D*···*A*	*D*—H···*A*
C353—H353···O1^i^	0.93	2.47	3.288 (4)	147
C12—H12···*Cg*3^ii^	0.93	2.93	3.761 (4)	150
C13—H13···*Cg*4^ii^	0.93	2.73	3.547 (4)	148

Symmetry codes: (i) −*x*+1, −*y*+1, −*z*; (ii) *x*−1, *y*, *z*.
